# Hydrothermal-based Wastewater Solids Management for Targeted Resource Recovery and Decarbonization in the Contiguous U.S.

**DOI:** 10.1021/acs.est.5c07190

**Published:** 2025-09-09

**Authors:** Jianan Feng, Timothy J. Strathmann, Jeremy S. Guest

**Affiliations:** 1 The Grainger College of Engineering, Department of Civil and Environmental Engineering, 14589 University of Illinois Urbana−Champaign, Urbana, Illinois 61801, United States; 2 Department of Civil and Environmental Engineering, 3557 Colorado School of Mines, Golden, Colorado 80401, United States; 3 Institute for Sustainability, Energy, and Environment, University of Illinois Urbana−Champaign, Urbana, Illinois 61801, United States

**Keywords:** Circular economy, decarbonization, defossilization, sludge, biosolids, techno-economic analysis (TEA), life cycle assessment (LCA), geospatial analysis

## Abstract

Wastewater solids management is a key contributor to the operational cost and greenhouse gas (GHG) emissions of water resource recovery facilities (WRRFs). This study proposes a ‘waste-to-energy’ strategy using a hydrothermal liquefaction (HTL)-based system to displace conventional energy- and emission-intensive practices. The proposed system directs HTL-produced biocrude to oil refineries and recovers regionally tailored nitrogen and phosphorus fertilizers. In an independent facility analysis, 576 WRRFs in the contiguous U.S. (CONUS) could deploy financially viable HTL-based wastewater solids management, simultaneously achieving cost savings of 4.81M [4.04M to 5.51M] $·day^–1^ and a GHG reduction of 1,300 [351 to 2,140] tonne CO_2_ eq·day^–1^ while offsetting ∼ 1 to 2% of synthetic fertilizers. Key sustainability drivers include the biochemical composition of solids and internal rate of return (IRR), though IRR becomes less impactful at larger WRRFs. In a hub analysis, shared processing of wastewater solids at HTL-based treatment centers expands financially driven decarbonization opportunities to WRRF networks with solids mass flow rates higher than 5 to 7 tonne·day^–1^ and average transportation distances less than 80 to 155 km. Overall, this study highlights the potential of HTL-based systems for financially viable wastewater solids management while reducing GHG emissions and achieving targeted resource recovery in the CONUS.

## Graphical abstract

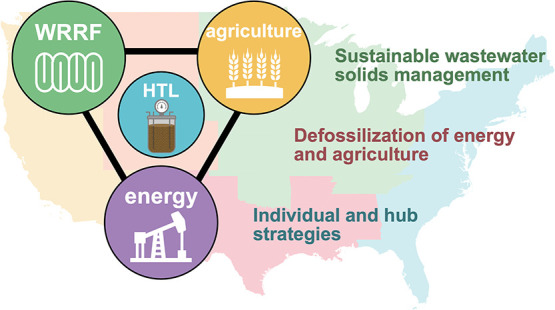

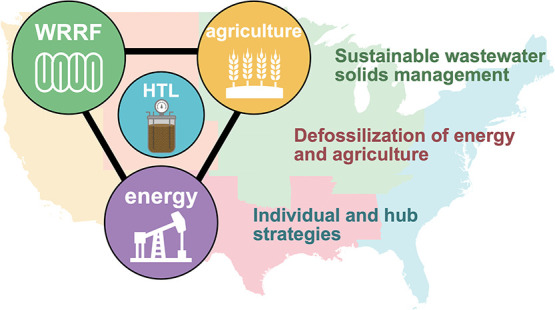

## Introduction

Conventional management practices for wastewater residual solids (i.e., sludge and biosolids) account for 20% to 60% of operational costs and up to 40% of the life cycle greenhouse gas (GHG) emissions from water resource recovery facilities (WRRFs).^1,2^ While the financial burdens of more advanced residual solids management can potentially be mitigated by selling value-added products (e.g., biosolids, biogas) and by leveraging tax credits and subsidies (e.g., the United States’ Inflation Reduction Act for biogas), simultaneously reducing the life cycle environmental impacts of residuals management remains a challenge.^3^ This is due to multiple emission sources during various management steps (i.e., treatment,^4–6^ storage,^7,8^ and disposal^9,10^ ) and the predominance of fugitive CH_4_ and N_2_O emissions, which are 29.8 and 273 times as potent as CO_2_, respectively, for global warming potential over a 100-year time horizon.^11^ For example, anaerobic digestion of sludge results in the fugitive release of (on average) 13.3 g CH_4_·m^–3^ wastewater, which is an order of magnitude higher than other unit operations at WRRFs.^5^ Additionally, land application of biosolids emits up to 1.8 kg N_2_O·tonne^–1^ biosolids,^12^ and its future is becoming increasingly uncertain due to the prevalence of emerging contaminants such as per- and polyfluoroalkyl substances (PFAS).^13,14^ Given the cost and environmental challenges associated with conventional practices for residual solids treatment and disposal, there is an urgent need for innovative solutions that can achieve safe and more sustainable wastewater solids management.

As an alternative to conventional treatment and disposal routes, thermochemical ‘waste-to-energy’ strategies capitalize on the energetic content of wastewater solids (i.e., 8 to 21 MJ·kg^–1^, on a dry weight basis)^15^ to produce marketable products while reducing the final residuals requiring disposal. One of the most promising ‘waste-to-energy’ technologies for wastewater solids management and valorization is hydrothermal liquefaction (HTL), which is particularly suitable for high-moisture-content waste streams and has the potential to mitigate 0.5 to 9.6 tonnes CO_2_ eq·tonne^–1^ feedstock.^16^ Recently, a HTL-based system has been demonstrated to effectively reduce both wastewater solids management cost and environmental impact by producing renewable diesel and fertilizers (ammonium sulfate and struvite).^17^ Multiple studies have also demonstrated the ability of HTL to eliminate contaminants embedded in wastewater solids (including antibiotics, microplastics, and various types of PFAS).^18–20^ Though promising, questions remain about the broad potential of HTL-based systems. For example, due to the high capital expenditure (CAPEX), it has been proposed that only WRRFs with sufficient scale (i.e., larger than 20 MGD) can economically implement this system.^17^ To overcome this challenge, HTL-derived biocrude could be coprocessed at existing, centralized oil refineries to prevent the need for onsite hydrotreating and hydrocracking.^21^ Co-processing is theoretically feasible if the blending ratio between biocrude and fossil-derived crude oil is low and could save more than 10% of the CAPEX to enable smaller WRRFs to benefit from HTL-based systems.^17^ Additionally, while HTL-based systems have been designed to recover nutrients in the form of ammonium sulfate and struvite, the market shares of these fertilizers are limited (7% and 0%, respectively, as of 2015) in the contiguous U.S. (CONUS).^22^ Instead, systems could be redesigned to align recovered nutrient products with regional demand, producing fertilizers in the form of anhydrous ammonia, urea, and urea ammonium nitrate (UAN) for nitrogen, and diammonium phosphate (DAP) for phosphorus.^22,23^

Despite these opportunities, existing studies have primarily focused on demonstrating the general feasibility of HTL-based systems, often relying on streamlined assumptions about deployment contexts and resource recovery pathways (e.g., targeting uncommon forms of fertilizers). This misalignment with the localized market demand undermines the model applicability to guide decision-making in specific settings. To fill this gap, analyses must incorporate detailed WRRF data (e.g., the mass flow rate of wastewater solids, wastewater solids biochemical compositions, incumbent management practices) and other localized contextual parameters (e.g., grid structure, income tax, labor cost) to generate site-specific insights. If achieved, these analyses could guide the development of HTL-based solutions with targeted resource recovery to align with existing energy infrastructure and local resource requirements, ensuring that wastewater solids-derived products can enter the market and offset conventional fossil-based resources (i.e., defossilization). Altogether, these tailored solutions could transform wastewater solids management into a more sustainable and integrated process.

The objectives of this work were (i) to characterize the potential for HTL-based systems to achieve financially viable carbon intensity (CI) reduction and resource recovery, and (ii) to elucidate contextual drivers governing the ability of HTL-based systems to advance sustainable wastewater solids management and clean resource transitions across the CONUS. An overview of the analytical framework and data flow is provided here, with details in the Methods section. First, an agile HTL-based system was developed in QSDsan^24,25^ and BioSTEAM^26,27^ (open-source modeling platforms; Section S3) with the ability to adapt configuration design and operation to align resource recovery with local/regional resource demands. In an independent facility analysis, it was assumed one HTL-based system serves one WRRF. This system routes biocrude for co-processing at existing oil refineries and produces nitrogen and phosphorus fertilizers tailored to surrounding agricultural practices. Then, to guide locality specific design decisions, an integrated geospatial database was established, encompassing more than 15,000 WRRFs, 114 oil refineries (i.e., all active oil refineries in the CONUS), regional agricultural fertilizer usage, and other context-specific parameters across the CONUS. This database facilitated automated configuration selection and detailed design of HTL-based systems based on local market conditions, as well as the identification of the biocrude transportation network to minimize transportation-related costs and environmental impacts. To further expand the opportunity space of HTL-based wastewater solids management for WRRFs facing economic scale limitations, a hub analysis was conducted where it was assumed centralized HTL-based hubs could enable shared wastewater solids processing. For both individual and hub analyses, techno-economic analysis (TEA) and life cycle assessment (LCA) were conducted to evaluate the financial implications and GHG reduction potential of HTL-based systems under uncertainty. Finally, at the national scale, the potential for HTL-based systems to reduce the reliance on traditional fossil-based crude oil and synthetic fertilizers was assessed. Overall, this study demonstrates the potential of HTL-based systems to advance sustainable waste management and to facilitate circular economy transitions for WRRFs within the CONUS.

## Methods

### Integrated database of WRRFs, fossil energy facilities, local resource demands, and other contextual parameters

An integrated database was built from various sources to facilitate model development and related analyses (Figure 1A). Specifically, a data set including 15,863 WRRFs were obtained from El Abbadi et al.^12^ Key WRRF attributes include facility locations, treatment capacity (measured as the design influent flow rate in million gallons per day, MGD), wastewater solids mass flow rates, incumbent wastewater solids management practices, itemized life cycle GHG emissions, and the configuration of treatment trains (e.g., the presence or absence of sludge digestion). A more detailed description of this database is included in Section S1.1. Information on 114 oil refineries (with nonzero production capacity; for biocrude upgrading; Figure S2A) and 191 coal-based power plants (with nonzero production capacity; for hydrochar cofiring; Figure S2B) in the CONUS were obtained from the U.S. Energy Information Administration.^28,29^ Additionally, county-level nitrogen and phosphorus fertilizer demand data were retrieved from the U.S. Geological Survey.^30^ The regional distribution of dominant fertilizer forms was derived from U.S. Department of Agriculture^22^ and Cao et al.^23^ Other contextual parameters included state-level electricity price,^31^ regional-level electricity carbon intensity (CI, a.k.a. global warming potential, GWP),^32^ state-level fertilizer price,^33^ state-level crude oil first purchasing price,^34^ state-level income tax,^35^ and county-level labor wage (annual average wage level for all industries owned by local government).^36^ Electricity CI data for 134 simulated regions were based on the NREL 2021 Standard Scenarios Cambium Data,^32^ which provides results comparable to the U.S. EPA eGRID data (including 68 balancing authorities), but includes emissions from additional sources (behind-the-meter photovoltaics, storage, and imports) and offers finer geospatial granularity.^37^ It should be noted the electricity-related emissions in the original WRRF database^12^ were based on NREL 2020 Standard Scenarios Cambium Data^38^; this discrepancy could have very minor impacts on the WRRF life cycle GHG reduction. All contextual data were obtained with the highest possible granularity. When any data were unavailable, regional or national average values were applied. All data are available in Sections S1 and S2, and/or the supporting Excel file.

**Figure 1:**
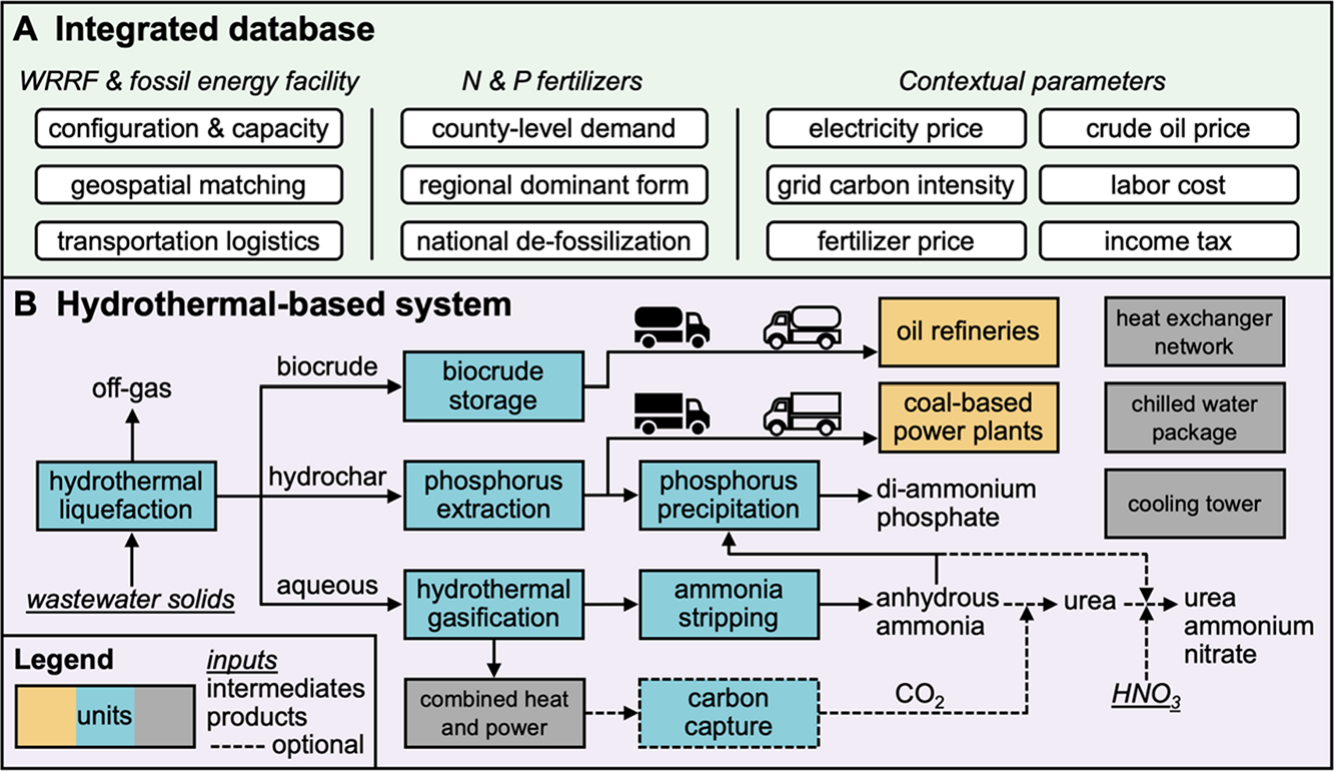
(A) Summary of the integrated database of WRRF and fossil energy facility information, nitrogen and phosphorus fertilizer demand, and contextual parameters considered during the simulation of HTL-based process models. (B) Flow diagram of HTL-based wastewater solids management systems. Units in blue and gray represent inside- and outside-battery limit units, respectively. Units and processes outlined in dashed lines represent alternative pathways for producing nitrogen fertilizers. Oil refineries and coal-based power plants are represented as yellow blocks and are outside the system boundary.

### HTL-based wastewater solids management system

An HTL-based wastewater solids management system (Figure 1B) was modeled using QSDsan^24,25^ and BioSTEAM.^26,27^ The moisture content of the residual wastewater solids has a minimal impact on the results and was assumed to be 80% for all WRRFs,^17^ but the biochemical compositions (i.e., ash, lipid, protein, and carbohydrate contents) were varied based on whether existing solids processing included anaerobic digestion, aerobic digestion, or no digestion (Table S3). Wastewater solids were pressurized to 21 MPa and were heated to 350 °C before entering the HTL reactor. In the HTL reactor, the wastewater solids were converted into biocrude, off-gas, hydrochar, and an aqueous phase with a 15 min retention time. The amount of HTL products and their compositions were estimated using a multiphase component additivity model with the biochemical composition of the wastewater solids as the input.^39–41^ The HTL products were separated through knockout drums (for gas venting), a solids filter, and a gravimetric oil/water separator.^42,43^ After phase separation, hydrochar was treated with 0.5 M H_2_SO_4_ at 1 g:7 mL to extract phosphorus. The remaining hydrochar was transported to coal-based power plants by trucks for cofiring. However, due to the expected low yield of hydrochar (e.g., < 5%, compared to >60% for biocrude)^17^ and the current unstable market of hydrochar,^44^ the main focus of this study was on biocrude production and nutrient recovery. Specifically, biocrude was stored and transported to oil refineries by tank trucks. The HTL aqueous phase product was further treated in a catalytic hydrothermal gasification (CHG) unit to mineralize and gasify the residual dissolved organics to fuel gases (approximately 70% v/v CH_4_).^43^ Ammonia was then stripped from the CHG effluent and reacted with phosphorus extracted from hydrochar to produce diammonium phosphate (DAP). Multiple nitrogen recovery pathways were designed to recover nitrogen fertilizers in different forms: (i) anhydrous ammonia, by dewatering stripped ammonia through molecular sieves and compressing dried ammonia in a compressor followed by cooling; (ii) urea, by reacting dried ammonia with CO_2_ captured from the flue gas of the combined heat and power (CHP) unit; and (iii) urea ammonium nitrate (UAN), by mixing urea (produced from dried ammonia and captured CO_2_) and ammonium nitrate (produced from dried ammonia and nitric acid solutions). HTL-based systems were designed to recover the most prevalent nitrogen fertilizer in each location (Table S1). On the energy side, the system included a heat exchanger network to offset part of the heating and cooling demand, with the remainder met by a CHP unit (which combusted the fuel gas from CHG and additional natural gas as needed), a chilled water package, and a cooling tower. Detailed descriptions of each unit were included in our previous work^17^ and Section S3. All related Python scripts can be accessed online.^45^

### System-level integration and transportation model

HTL-produced biocrude has been demonstrated to be resistant to degradation.^46,47^ Therefore, it has the potential to be transported to oil refineries for further refinement with minimal in-transit degradation. Additionally, it was assumed all included oil refineries have sufficient spare capacity to process the additional HTL-derived biocrude, given the anticipated low biocrude-to-crude-oil ratio (demonstrated to be <0.01% in the individual strategy). In this work, the geospatial coordination information on 15,863 WRRFs^12^ (Figure S1) and 114 oil refineries^28^ (Figure S2A) in the CONUS were integrated into the Web Mercator Coordinate system and were matched based on their nearest linear distances. Actual travel distances between matched facilities were then calculated using the Google Maps API under the ‘driving’ mode.^48,49^ Eight WRRFs were excluded in this step as they cannot be reached by driving (Section S3.2). It should be noted the transportation logistics in this study was limited to the CONUS and the results from this simplified geospatial assessment should only be taken as first-order estimates of the WRRF-oil refinery collaboration. Contextual rules and business models that may affect such collaborations were out of the scope of this work and could be addressed in future context-specific studies. It was assumed biocrude was purchased from WRRFs and that the wastewater utilities were also responsible for the transportation of biocrude through tank trucks. The cost of transportation was composed of fixed cost (e.g., for loading and unloading biocrude; 7.60 $·m^–3^, 2022 U.S. dollars throughout this study) and variable cost which was proportional to the one-way transportation distance (0.09 $·m^–3^·km^–1^).^50^ In addition, the environmental impacts of transportation via tank trucks were estimated with a baseline CI of 0.13 kg CO_2_ eq·tonne^–1^·km^–1^, where distance is based on one-way transportation but the value includes the impact of empty return trips as well.^51^ A similar transportation model was developed for hydrochar, with detailed cost and CI data in Table S8 and Table S6, respectively.

### HTL-based hub analysis for shared wastewater solids processing across WRRFs

To explore potential implementation strategies that could support the use of HTL-based treatment systems at more WRRFs, the potential for shared wastewater solids processing was evaluated. This approach would enable smaller-scale WRRFs to consolidate wastewater solids at centralized HTL-based hubs, thus enhancing both financial viability and environmental benefits by taking advantage of economies of scale. In this scenario, the economic and environmental implications of transporting wastewater solids to centralized HTL-based hubs were evaluated across varying combinations of total wastewater solids mass flow rates and average solids transportation distances. A conservative assumption for this analysis was that all wastewater solids have a high ash content (similar to postdigestion solids). The baseline transportation cost comprises both fixed cost (5.53 $·m^–3^) and variable cost (0.05 $·m^–3^·km^–1^; based on one-way transportation distance),^52^ and the baseline CI of trucking is 0.13 kg CO_2_ eq·tonne^–1^·km^–1^ (based on one-way transportation distance, but with the CI value accounting for empty return trips).^51^ Wastewater solids decomposition was assumed to be minimal during transport from participating WRRFs to HTL-based hubs, given the expected short transportation times. Three cases targeting different nitrogen fertilizers were evaluated. Based on the model simulation results (Figure 5, Figure S13–S15), two constraints were identified, including a minimum wastewater solids mass flow rate of 7 tonne·day^–1^ to achieve financial viability and a maximum allowable average transportation distance between participating WRRFs and HTL-based hubs of 80 km to ensure net GHG reductions. Both constraints were selected to be conservative to minimize false positives. The transportation distance constraint was converted to the linear distance constraint by dividing it by a constant factor of 1.3 (Section S6, Table S15). Next, under these constrains, candidate HTL-based processing hubs were identified using k-d tree-based nearest neighbor search and graph algorithms (Section S6). As the financial viability and GHG reduction potential of these HTL-based hubs have been established, the focus was on estimating additional resource recovery potential. This was accomplished by scaling baseline yields of biocrude, nitrogen fertilizer, and phosphorus fertilizer from wastewater solids based on the aggregated solids mass flow rate from all participating WRRFs.

### Techno-economic analysis (TEA) and life cycle assessment (LCA)

TEA and LCA were performed to evaluate the financial feasibility and GHG reduction potential of HTL-based systems for WRRFs displacing conventional wastewater solids management practices (i.e., landfilling, land application, and incineration; Table S4). TEA was conducted through a 30-year cash flow rate of return analysis to assess the net savings or additional expenses. A previous study reported different ranges for the internal rate of return (IRR; the discount rate at which the net present value equals zero, i.e., the system breaks even) and the interest rate (the rate paid on a loan) for HTL-based systems from the perspective of either a waste management entity or a biofuel producer.^17^ In this study, HTL-based systems were primarily designed for wastewater solids management (but not for profit); therefore, lower IRR and interest rate values (both with a baseline value of 3%) were used. All costs in this study were converted to 2022 U.S. dollars using the Gross Domestic Product: Chain-type Price Index,^53^ the Chemical Engineering Plant Cost Index,^54^ and the Employment Cost Index.^55^ A complete list of assumptions for TEA can be found in Table S5. For LCA, the Ecoinvent v3.8 database (the cutoff model)^51^ was used for life cycle inventory data and the U.S. EPA’s Tool for the Reduction and Assessment of Chemicals and Other Environmental Impacts (TRACI)^56^ was used for life cycle impact assessment, with a focus on CI (Table S6). Impacts were allocated using the system expansion method,^57^ with recovered products displacing fossil-derived products in the market. Other impact categories (e.g., acidification, ozone depletion) were considered in Feng et al.^17^ for HTL-based systems but were not considered in this study due to the absence of facility-level baseline data from conventional wastewater solids management. It should be noted construction has been demonstrated to account for less than 2% of the life cycle GHG emission of HTL-based systems (Table S7).^17^ Therefore, it was excluded from the LCA in this study, which was also consistent with current policy practices (e.g., the Renewable Fuel Standard,^58^ the 45Z tax credit^59^). Additionally, the carbon in wastewater solids was treated as biogenic, and the resulting CO_2_ emissions (directly derived from the organic carbon) were assumed to be climate-neutral (i.e., not counted toward the CI).

### Uncertainty and sensitivity analyses

During the initial round of the individual facility analysis (including model simulations, TEA, and LCA), uncertainty analysis was excluded due to the large number of WRRFs (i.e., 15,855). Instead, baseline results were used to select WRRFs with net positive financial savings and GHG reductions when switching to HTL-based wastewater solids management systems. For these candidate WRRFs, Monte Carlo Simulations with Latin Hypercube Sampling (N = 1,000) were conducted to capture the technological and deployment-related uncertainties associated with HTL-based systems (a complete list of uncertain parameters and their distributions can be found in Table S8). Facilities yielding incomplete or invalid uncertain results due to extreme parameter combinations were excluded from the candidate WRRF list to ensure the consistent performance of HTL-based systems. This led to the exclusion of one WRRF in this study. When aggregating facility-level results, Latin Hypercube Sampling with different Gaussian copula correlation coefficients was tested and a coefficient of 0.8 was selected to reflect the strong correlation among the facility-level results due to shared parameters (e.g., market conditions, technology assumptions; Section S5.2, Table S13). For the sensitivity analysis, to cover the full range of wastewater solids mass flow rates, 15,855 WRRFs (regardless of their baseline results) were first classified into 1,000 groups using an unsupervised machine learning method—Agglomerative Hierarchical Clustering (the total group number of 1,000 was selected to balance the result representativeness and the computational burden; Section S5.3). Then, median values of contextual parameters in each group were used to generate reconstituted WRRFs data. For each reconstituted WRRF, Spearman’s correlation coefficients were calculated based on the Monte Carlo Simulation results (with Latin Hypercube Sampling, N = 1,000; required sample size determined using the Fisher’s Z-test with Bonferroni correction; Section S5.4). Key sustainability drivers were defined as parameters with a Spearman’s p-value less than 0.05 and an absolute Spearman’s ρ-value higher than 0.2 toward wastewater solids management cost or CI for at least 10% of reconstituted WRRFs.

## Results and Discussion

### Prioritizing standalone WRRFs deploying HTL-based systems

#### Identifying candidate WRRFs

In the independent facility analysis, HTL-based systems were assumed to be deployed at individual WRRFs. Among the 15,863 WRRFs analyzed in this study, 576 facilities were identified as candidate sites for transitioning to HTL-based systems for both cost and GHG reductions (Figure 2A). The remaining facilities were excluded primarily due to their failure to reduce cost (Table S9). Notably, these 576 candidate facilities account for 52.6% of total wastewater residual solids and 53.0% of existing GHG emissions from WRRFs across the CONUS. A full list of these WRRFs can be found in the supporting Excel file. Replacing conventional wastewater solids management with HTL-based systems at these select facilities is projected to achieve potential cost savings ranging from 49.4 [−473 to 635] $·day^–1^ (median value followed by 5th to 95th percentiles in brackets) to 166,000 [152,000 to 182,000] $·day^–1^. GHG reductions span from –0.764 [−2.93 to 1.14] tonne CO_2_ eq·day^–1^ (the baseline GHG reduction without uncertainty for this facility was 0.120 tonne CO_2_ eq·day^–1^) to 38.0 [22.3 to 51.9] tonne CO_2_ eq·day^–1^, where a positive value indicates a net reduction in life cycle GHG emissions and a negative value indicates a net increase. These reductions correspond to an estimated 61.5% [35.8% to 99.4%] of wastewater solids management-related GHG emissions and 2.6% [0.8% to 5.9%] of total WRRF life cycle emissions (Figure 2B). The GHG reduction can be largely attributed to the recovery of biofuels and fertilizers, as well as the production of electricity (Table S11). Notably, the potential GHG reductions could be achieved in parallel with net cost savings, making the transition to HTL-based wastewater solids management a compelling economic opportunity—particularly for WRRFs facing financial constraints. However, it is important to note the solids management emissions from the original WRRF data set only included disposal-related emission (e.g., fugitive emissions from landfills), whereas all other solids management processes were aggregated with mainline wastewater treatment due to the lack of detailed facility-level data on solids management unit processes.^12^ Additionally, it also excluded solids hauling-related emissions, which are expected to be relatively low (Figure S7). As a result, the actual GHG reduction percentage from solids management are likely to be higher than reported (Figure 2B, left panel). To improve future assessments, national surveys (e.g., the Clean Watersheds Needs Survey) could incorporate more detailed facility-level solids handling process data.

**Figure 2:**
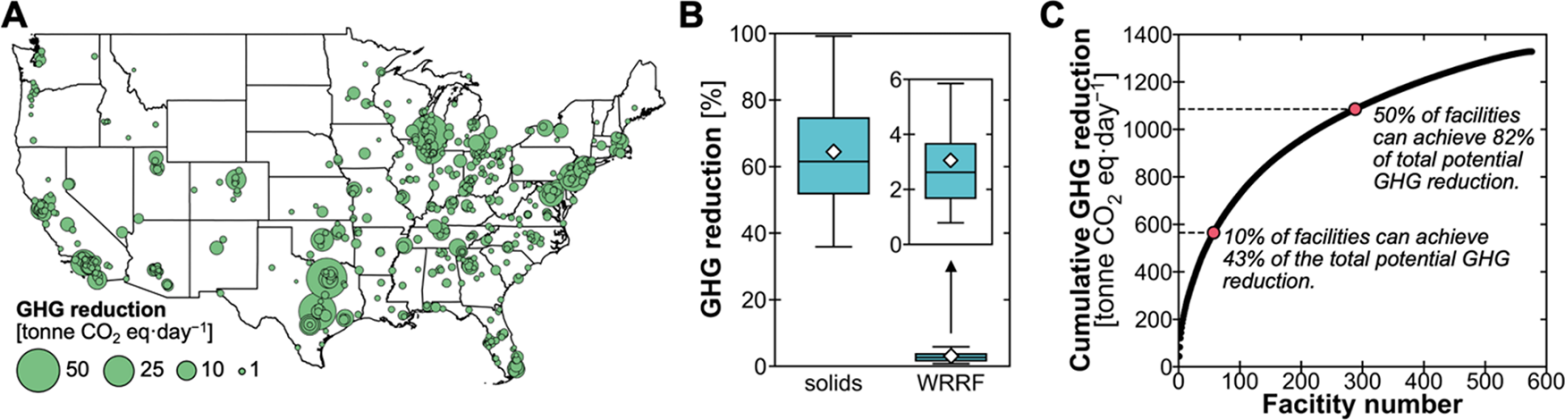
(A) A U.S. map showing all 576 WRRFs that have the potential to achieve financially viable GHG reduction by implementing HTL-based systems. The legends from left to right represent GHG reduction amount of 50, 25, 10, and 1 tonne CO_2_ eq·day^–1^, respectively. (B) Operational GHG reduction percentage of the solids management process across the identified 576 WRRFs. Whiskers, boxes, and midlines of box plots represent 5th and 95th percentiles, 25th to 75th percentiles, and 50th percentiles, respectively. The diamonds represent the average results. (C) Cumulative GHG reduction of identified 576 WRRFs. Facilities are sorted based on their GHG reduction potential, from highest reduction potential (low facility number) to lowest reduction potential (high facility number).

Due to economies of scale, the primary driver of wastewater solids management unit costs (i.e., $·tonne^–1^) in HTL-based systems is the mass flow rate of solids processed (Figure S9A, Table S12). Net costs are also dependent on the avoided costs of the existing conventional disposal method (Table S4), among which land application yields the highest avoided cost (606 $·tonne^–1^), followed by incineration (441 $·tonne^–1^) and landfilling (413 $·tonne^–1^). From an environmental perspective, the CI of wastewater solids management (kg CO_2_ eq·tonne^–1^) is less dependent on the mass flow rate of wastewater solids processed (Figure S9B, Table S12); instead, the biocrude transportation distance, the hydrochar transportation distance, the wastewater solids biochemical compositions, and the type of recovered nitrogen fertilizers have stronger influences (Figure S9D, F, H, Table S12). Final GHG reductions are also affected by the avoided GHG emission from existing management practices (Table S4), including land application (producing N_2_O at 210 kg CO_2_ eq·tonne^–1^), landfilling (producing CH_4_ at 168 kg CO_2_ eq·tonne^–1^), and incineration (no nonbiogenic emission). From a cumulative GHG reduction perspective across WRRFs (Figure 2C), a total of 1,330 tonne CO_2_ eq·day^–1^ (the sum of baseline results of each candidate WRRF; the result considering uncertainties is 1,300 [351 to 2,140] tonne CO_2_ eq·day^–1^) GHG emission could be reduced in the CONUS—with the top 10% of candidate WRRFs accounting for 43% of total potential reductions, and the top 50% contributing to 82% of potential reductions—suggesting prioritizing deployment at these high-impact facilities could maximize environmental benefits in the near-term. More importantly, such decarbonization benefit is driven by a simultaneous 4.81M [4.04M to 5.51M] $·day^–1^ nationwide cost saving. It should be noted the values of avoided cost and CI (from conventional residual solids disposal) used in this study reflect general CONUS-wide assumptions due to the lack of facility- and regional-level data (e.g., fugitive emissions from landfills). To mitigate this limitation, the absolute wastewater solids management cost and CI associated with all simulated HTL-based systems have been included in the Supporting Excel file for future facility-specific evaluations.

To provide regional insights on HTL-based systems as a cost-effective way for climate change mitigation, the CONUS is divided into five regions: East Coast, Midwest, Gulf Coast, Rocky Mountain, and West Coast. Geographically, candidate WRRFs are concentrated in the New England area, the Great Lakes region, California, and Texas (Figure 2A). The East Coast demonstrates the highest GHG reduction potential (395 [112 to 675] tonne CO_2_ eq·day^–1^; Figure S10) and the second highest cost reduction (1.45M [1.21M to 1.70M] $·day^–1^). This is attributed to the highest density of large WRRFs, particularly in the Mid-Atlantic subregion. The Midwest achieves a similar GHG reduction (380 [80.4 to 673] tonne CO_2_ eq·day^–1^) but has higher cost savings (1.47M [1.25M to 1.68M] $·day^–1^) than the East Coast, largely due to the prevalence of land application in the Midwest which yields higher avoided costs. However, most of these contributions come from Great Lakes states including Ohio, Indiana, Michigan, Illinois, Wisconsin, and Minnesota. In contrast, individual WRRFs in other Midwest states (e.g., North Dakota, South Dakota) often lack the economies of scale to make the deployment of HTL-based systems financially feasible. The West Coast and the Gulf Cost achieve moderate cost savings and GHG reductions, due to the presence of several large WRRFs in California and Texas. As a result, the West Coast has the potential to save an estimated 0.975M [0.825M to 1.15M] $·day^–1^ and reduce GHG emissions by 237 [83.4 to 405] tonne CO_2_ eq·day^–1^, while the Gulf Coast could save 0.770M [0.659M to 0.879M] $·day^–1^ and reduce GHG emissions by 232 [80.2 to 379] tonne CO_2_ eq·day^–1^. Finally, the Rocky Mountain region, dominated by smaller WRRFs, shows the lowest potential savings (0.132M [0.108M to 0.158M] $·day^–1^) and GHG reductions (44.7 [11.2 to 82.7] tonne CO_2_ eq·day^–1^), which are negligible compared to other regions. Overall, these findings highlight the substantial yet regionally distinct potential of HTL-based systems, driven primarily by the uneven geographic distribution of large-sized WRRFs. This variation underscores the need for technology developers and design firms to strategically prioritize the deployment of HTL-based systems at individual WRRFs across the CONUS.

#### Fossil-based resources offset

The implementation of HTL-based wastewater solids management at 576 candidate WRRFs also contributes to local defossilization by recovering valuable resources. Specifically, HTL-produced biocrude can be transported to nearby oil refineries with feasible transportation distances (203 [36.0 to 589] km for all 15,855 WRRFs included in the analysis, Figure S8). At the facility-level, biocrude production ranges from 8.69 [7.15 to 10.4] barrel·day^–1^ (BPD) to 443 [369 to 528] BPD (where one barrel is equivalent to 42 gallons or 159 L). Compared to onsite biocrude upgrading at WRRFs through hydrotreating and hydrocracking, integration with existing oil refineries not only enhances the market accessibility of oil products, but also reduces economic burdens for wastewater utilities (Table S10) and significantly lowers the minimum required size of facilities for financial viability from around 20 tonne·day^–1^ (from Feng et al.^17^) to 5.0 tonne·day^–1^ (this work). At the state-level, California, Texas, and Florida emerge as top wastewater-derived biocrude producers, with median generations of 2,910 BPD, 2,420 BPD, and 1,340 BPD, respectively (Figure 3A). Some states (e.g., New Jersey, Alabama, Illinois) have the potential to receive substantial biocrude imports from neighboring states, despite lower in-state production. In most cases, biocrude could be transported within the state, as seen in California and Texas, or at least within the same Petroleum Administration for Defense Districts (PADD) region (e.g., New York to New Jersey, Wisconsin to Illinois; Figure 3A). Only in rare cases does transportation cross PADD regions, such as Florida to Alabama. This pattern demonstrates high logistical efficiency, minimizing transportation-related costs and environmental impacts. Such advantages could be further leveraged as WRRFs become local integration points for other types of feedstocks (e.g., microalgae, food waste, animal waste) for centralized HTL-based valorization.

**Figure 3:**
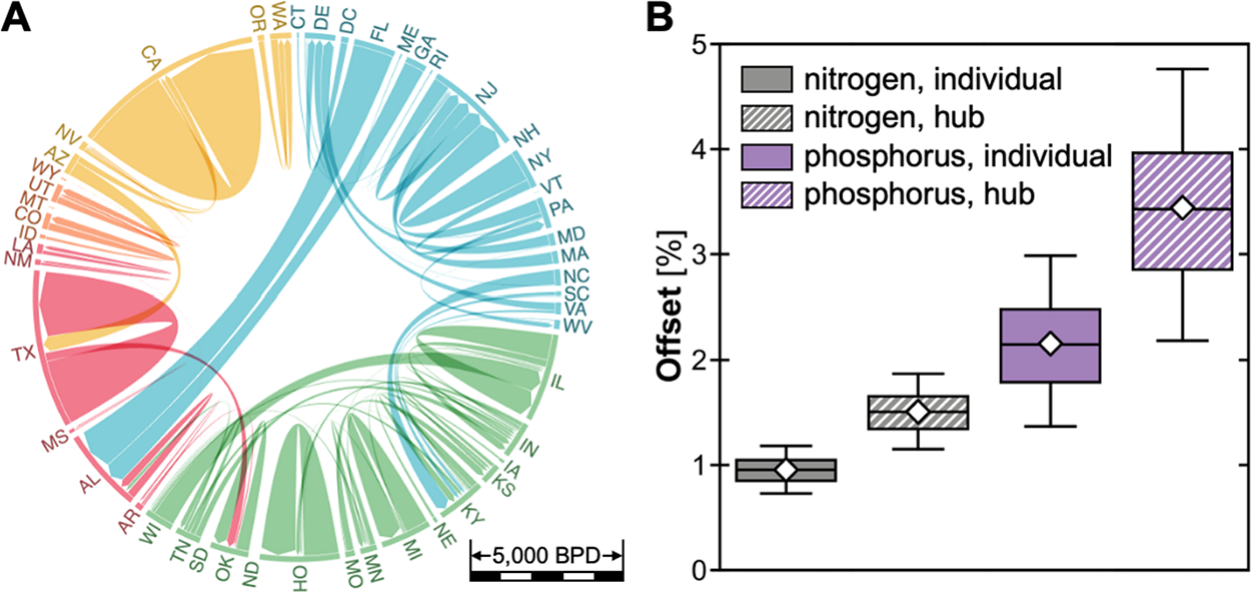
(A) Biocrude transportation across states. Arrows begin from the edge of the circle where biocrude is produced and point to the state where it would be transported for processing. Colors represent different regions of the contiguous U.S. (CONUS; blue: East Coast, green: Midwest, red: Gulf Coast, yellow: West Coast, orange: Rocky Mountain), and width of the connections is proportional to biocrude flow. The full length of the scale bar represent biocrude flow rate of 5,000 barrel·day^–1^ (BPD). (B) The percentage of conventional synthetic fertilizers that could be offset by nitrogen and phosphorus fertilizers yielded from HTL-based systems in the CONUS. Gray represents nitrogen and purple represents phosphorus. Solid color fill represents the individual WRRF scenario (i.e., only including 576 WRRFs as shown in Figure 2A) while the pattern fill represents the hub scenario (i.e., including 5,692 potentially participating WRRFs in the CONUS). Whiskers, boxes, and midlines of box plots represent 5th and 95th percentiles, 25th to 75th percentiles, and 50th percentiles, respectively. The diamonds represent the average results.

With regard to nutrient circularity, resource recovery can be tailored to local agricultural practices. For example, nitrogen is assumed to be recovered as anhydrous ammonia, urea, or UAN, depending on the dominant form used in the region (Table S1). Phosphorus is consistently recovered as DAP, the primary phosphorus fertilizer used nationwide. Compared to other resource recovery strategies, such as struvite precipitation, producing fertilizers with an established market offers more facile integration with existing supply chains and enhanced scalability. Additionally, the recovery and sale of fertilizer products provide financial incentives to WRRFs, aligning environmental and economic goals for individual facilities. Based on 2017 fertilizer demand data, HTL-based wastewater valorization could potentially offset more than 10% of local synthetic nitrogen and phosphorus fertilizers (based on median results) for 152 and 221 counties, respectively (Figure S11). However, when aggregated across the CONUS, the offset potential is much lower—only 1.0% [0.7% to 1.2%] for synthetic nitrogen and 2.1% [1.4% to 3.0%] for synthetic phosphorus fertilizers (Figure 3B). These relatively low offset rates highlight the need for complementary strategies to support broader nutrient circularity, including coprocessing of other feedstocks (discussed above and below). Additionally, unleashing the full resource recovery potential from wastewater solids is also critical, as the individual scenario only taps into roughly half of the national available wastewater solids resources. Expanding the opportunity space for HTL-based wastewater solids management will require identifying key sustainability drivers that currently limit broader adoption.

#### Contextual drivers of the system-level sustainability

Feng et al. discussed some key technological advancements needed for the future research and development of HTL-based systems.^17^ In the current work, contextual parameters (i.e., parameters with location- and/or scenario-specific values) were investigated to prioritize deployment opportunities for the system. Some contextual parameters vary substantively across WRRFs but exhibit no or negligible variation for a given WRRF (e.g., the type of targeted nitrogen fertilizer, the mass flow rate of wastewater solids). Other contextual parameters vary less across facilities relative to their intrinsic uncertain ranges (e.g., crude oil price in Illinois).

For contextual parameters varying only between facilities, facility-level baseline results are compiled to assess their impacts on the system-level sustainability. These parameters include the mass flow rate of wastewater solids, the transportation distance of biocrude, the transportation distance of hydrochar, the presence or absence of wastewater solids digestion, and the type of recovered nitrogen fertilizer. The mass flow rate of wastewater solids is closely linked to the ultimate financial viability of HTL-based systems. A previous study reported HTL-based systems are capital intensive and the total CAPEX follows a power-function curve as the scale increases.^17^ In the current study, the mass flow rate of wastewater solids exerts the strongest influence on the unit costs of wastewater solids management (Figure S9A, Table S12) due to economies of scale. The minimum production rate of wastewater solids to support a financially viable HTL-based system is 5.0 tonne·day^–1^, and the likelihood of viability increased with increasing facility size (e.g., 55% of plants sized at 5 to 10 tonne·day^–1^ and 79% of plants larger than 50 tonne·day^–1^). However, WRRF size demonstrates less of an impact on the CI of the wastewater solids management (Figure S9B, Table S12). Biocrude and hydrochar transportation distances have minimal impacts on management cost (Figure S9C, Table S12) though as distances increase, slight increases in life cycle GHG emissions are observed (Figure S9D, Table S12). These findings indicate the geospatial relationship between WRRFs and fossil energy facilities (i.e., oil refineries and coal-based power plants) is generally not a limiting factor for the financially viable implementation of HTL-based systems in the CONUS. In addition, digestion alters the biochemical composition of wastewater solids, such as increasing ash content while reducing lipid content (Table S3).^60,61^ Three scenarios were considered in this work: aerobic digestion, anaerobic digestion, and no digestion. Aerobic and anaerobic digestion were assumed to yield biosolids with similar biochemical compositions, with the only difference being aerobic digestion leading to a 2.2% higher ash content.^60^ As a result, in this analysis, results from both digestion methods are not significantly different and are therefore combined. As expected, the reduced lipid content of residual solids after digestion lowers biocrude yield, increasing wastewater solids management GHG emissions but having relatively small impact on wastewater solids management cost compared to wastewater solids mass flow rate (Figure S9E-F, Table S12). Finally, the type of recovered nitrogen fertilizer has a relatively small impact, with anhydrous ammonia and UAN having better economic and environmental performance than recovering urea (Figure S9G-H, Table S12). Overall, wastewater solids mass flow rate is identified as the main deterministic factor controlling the financial feasibility of HTL-based systems at WRRFs across locations within the CONUS. However, for an individual WRRF (with relatively fixed wastewater solids mass flow rate), optimizing other contextual parameters—particularly those subject to higher intrinsic uncertainties (e.g., crude oil price, IRR)—could still benefit system-level sustainability.

To better understand the impacts of individual parameters on the economic and environmental sustainability of HTL-based system deployment at individual WRRFs, Spearman’s correlation coefficients were calculated for 1,000 reconstituted WRRFs (see Methods) with varying wastewater solids mass flow rates (0.00560 tonne·day^–1^ to 245 tonne·day^–1^). On the environmental side, the influences (i.e., Spearman’s ρ) of different parameters on the wastewater solids management CI does not show correlation with the wastewater solids mass flow rate (Figure S12) since CI is largely determined by the chemical and energy inputs per unit mass of wastewater solids managed and their associated emission factors, all of which remain relatively constant across WRRF sizes. In contrast to environmental impacts, the relative importance of select parameters on solids management cost are sensitive to the wastewater solids mass flow rate (Figure 4): this is because capital costs are an important financial driver and they exhibited economies of scale. For instance, the influence of biochemical compositions of wastewater solids (e.g., ash content, protein content) and crude oil price (which is directly related to the biocrude price) becomes significant as the mass flow rate of wastewater solids exceeds approximately 1 tonne·day^–1^, and continue to grow to become the most influential contextual drivers at wastewater solids mass flow rates above 10 tonne·day^–1^. Conversely, IRR, which represents the discount rate when the system breaks even, remains the strongest cost driver until the solids mass flow rate reaches 10 tonne·day^–1^, after which it is less influential. These observations suggest different financial strategies for WRRFs at varying economic scale. For example, facilities processing more than 10 tonne·day^–1^ of wastewater solids could optimize the biochemical composition of feedstocks such as through deashing or co-processing with higher-protein feedstocks. Additionally, these facilities may negotiate with oil refineries for higher biocrude first purchase prices. However, for smaller WRRFs, the best opportunity to reduce costs would be to secure lower interest financing (lowering IRR), for instance, through state and federal grants and loans for infrastructure upgrades. However, due to economic scale limitations, even at a 0% IRR, facilities with wastewater solids less than ∼ 10 tonne·day^–1^ are still not financially viable (Table S14). Therefore, a critical strategy moving forward would be to increase the wastewater solids processing capacity of deployed HTL-based systems. Scaling up processing not only leverages economic scale benefits but also shifts IRR to be a less critical factor compared to than other more tunable parameters (Figure 4). Based on this observation, integrating wastewater solids for larger-scale shared processing across WRRFs (i.e., the hub deployment strategy) is proposed to expand the deployment opportunities of HTL-based wastewater solids management systems.

**Figure 4:**
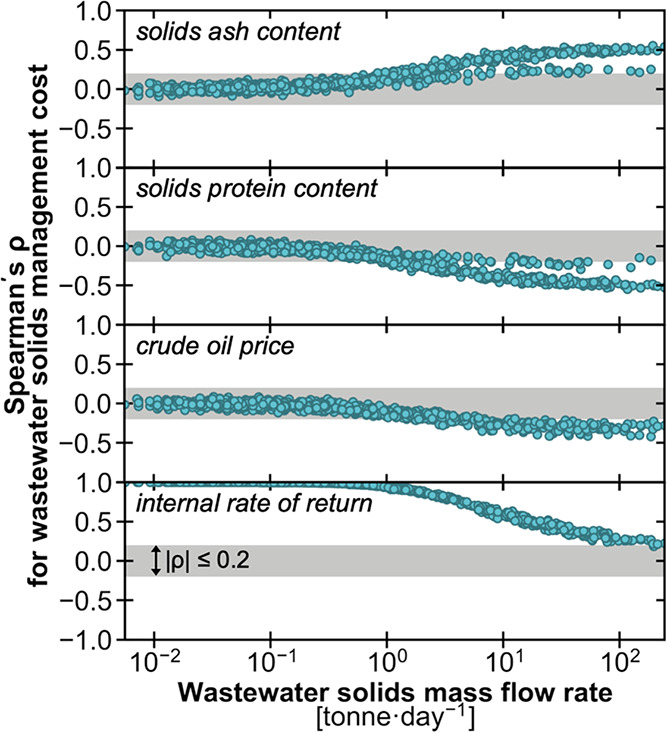
Spearman’s ρ value of selected contextual parameters for 1,000 reconstituted WRRFs with different sizes (blue circles) on wastewater solids management cost. Parameters are selected if Spearman’s *p* < 0.05 and the absolute value of Spearman’s ρ > 0.2 for at least 10% of reconstituted WRRFs. Each Spearman’s test is conducted based on 1,000 Monte Carlo simulations. Shaded areas represent Spearman’s ρ within ± 0.2.

### Expanding coverage through HTL-based hubs

#### Identifying candidate hubs

Centralized ‘hub’ HTL-based systems that integrate wastewater solids processing across multiple WRRFs represents a potential pathway to expand the opportunity space of HTL-based wastewater solids management, contingent on a more mature supply chain to support wastewater solids transportation. Given the extensive number of WRRFs in the CONUS, this study conducted a generalized analysis rather than proposing facility-specific solutions. For the case of UAN (the most commonly recovered nitrogen fertilizer), financial viability is achieved when the minimum solids mass flow rate reaches 5.8 tonne·day^–1^ at an average travel distance is 20 km, increasing slightly to 6.4 tonne·day^–1^ at 200 km (Figure S13A). This further underscores the finding that solids mass flow rate, rather than transportation distance, is the primary determinant of cost-effective HTL-based wastewater solids management. In contrast, GHG reductions are influenced by both wastewater solids mass flow rate and transportation distance (Figure S10B, Figure 5B). Beyond a threshold transportation distance of 125 km to 155 km (depending on the total solids mass flow rate), emissions associated with wastewater solids transportation become dominant, negating the GHG reduction benefits of resource recovery. This suggests that while transportation plays a less critical role in cost savings which is due to the capital-intensive nature of HTL-based systems, its impact on GHG reductions is more pronounced. This is, in large part, because transportation can represent a major source of operational emissions (e.g., up to 30%). To reduce the tension between the benefits of increasing mass flow rates and detriments of increasing transport distances, future research should explore more efficient and environmentally friendly transportation methods, such as railways, to mitigate GHG emissions further. Additionally, cases involving the recovery of anhydrous ammonia (Figure S14) and urea (Figure S15) follow similar trends, with slight shifts in the minimum solids mass flow rate required for net savings and the threshold transportation distance for net GHG reductions.

**Figure 5:**
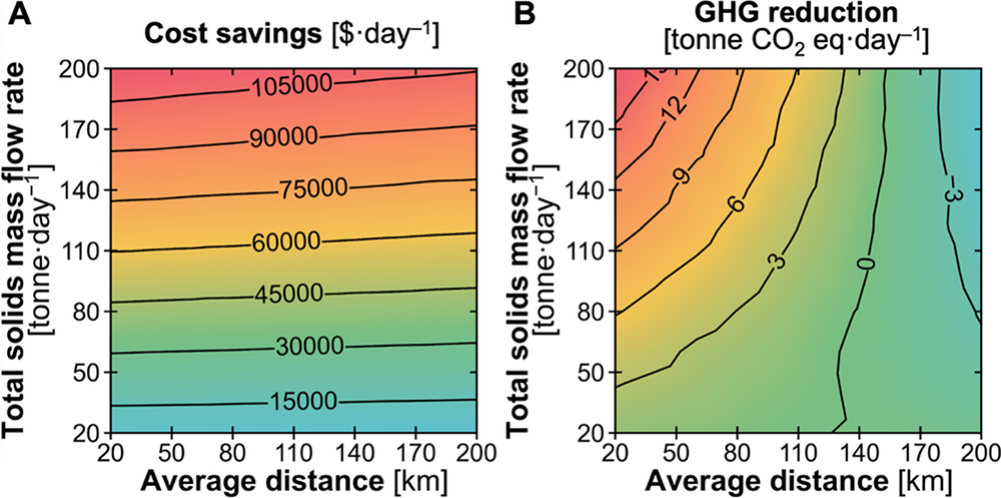
(A) Cost savings and (B) GHG reduction achieved by implementing HTL-based wastewater solids management systems (recovering urea ammonium nitrate, UAN) for 100 evenly distributed combinations of total wastewater solids mass flow rate (from 20 to 200 tonne·day^–1^) and average wastewater solids transportation distance (from 20 to 200 km). Each mass flow rate-distance combination was simulated with 1,000 Monte Carlo simulations, with median values shown in the figure.

In real-world implementations, the spatial configuration of wastewater solids transportation networks can vary significantly. For example, in major urban centers, multiple smaller WRRFs typically surround a larger central facility near high-population-density areas. In such cases, HTL-based systems could be located within the central WRRF to reduce the cost and environmental impact of wastewater solids transportation. Conversely, remote locations such as the Rocky Mountain region and part of the Midwest region consist of smaller, sparsely distributed WRRFs. Unlike the previous configuration, hubs in these regions can operate independently from WRRFs, allowing their locations to be optimized to minimize overall costs and GHG emissions from both wastewater solids and biocrude transportation. What’s more, some WRRFs have already established collaborative frameworks for wastewater solids management: for instance, some facilities truck residual solids to a separate WRRF for shared processing. For these facilities, transitioning to HTL-based systems represent a low­(er)-hanging opportunity to achieve more sustainable wastewater solids management and valorization. Though this study does not provide facility-specific implementation plans, it demonstrates HTL-based hubs could potentially achieve financially viable management of 84% of all wastewater solids streams from 5,692 WRRFs across the CONUS through 329 HTL-based hubs (261 hubs include more than one participating WRRF; Figure S16, Figure S17). Of the 5,692 WRRFs included, 5,153 facilities are uniquely included under the hub strategy (i.e., not covered by the individual strategy) and account for 33% of CONUS wastewater solids streams. Notably, more than 95% of these 5,153 WRRFs have wastewater solids mass flow rates below 5.0 tonne·day^–1^, which is the minimum value observed for feasible standalone deployment of HTL-based systems. This demonstrates expanded economies of scale benefits from aggregating wastewater solids for shared processing in HTL-based hubs. Future works should consider regional optimization strategies, including geospatial analyses to identify ideal hub locations, infrastructure needs for large-scale wastewater solids transportation, and policy incentives to encourage WRRF participation. Additionally, incorporating other organic waste streams (e.g., food waste, animal waste) could support the viability of new hub locations, which would enable the inclusion of more WRRFs by increasing overall feedstock availability.

#### National fossil-based resources offset

Compared to the individual strategy, HTL-based hubs could further expand defossilization of conventional energy and fertilizer sectors. Specifically, biocrude production could increase from 21,600 [18,100 to 24,900] BPD to around 35,000 BPD. This not only contributes further to the sustainability transitions of wastewater solids management, but also provides additional opportunities for clean fuel (e.g., renewable diesel) production and tax credits (e.g., via the Clean Fuel Production Credit).^62^ Such benefits could further incentivize the collaboration between WRRFs and oil refineries to enhance the viability of HTL-based systems by creating a self-sustaining loop where wastewater solids are continuously transformed into valuable commodities. Moreover, the offset of conventional synthetic nitrogen and phosphorus fertilizers increases through HTL-based hubs, with the estimated offset of 1.5% [1.1% to 1.9%] and 3.4% [2.2% to 4.8%], respectively. Currently, in the U.S., ammonia (a key precursor of nitrogen fertilizers) is primarily produced using the energy- and emission-intensive Haber-Bosch process.^63^ In addition, conventional phosphorus fertilizer production relies on regionally concentrated, nonrenewable supplies of phosphate rocks.^63^ However, the portion of these fossil-based resources ending up in wastewater solids are either incinerated, landfilled or suboptimally reused through land application. In contrast, HTL-based systems could facilitate the targeted resource recovery both to reduce the reliance on conventional fossil-based synthetic fertilizers and to support resource-resilient agriculture.

## Opportunities and Challenges

Transitioning wastewater solids management to HTL-based systems has the potential to displace conventional cost- and emission-intensive practices with a financially viable, carbon-neutral waste-to-energy system, thereby contributing to a more resource-secure future. In this study, we developed a first-order approximation of the potential of HTL-based systems to support both sustainable wastewater solids management and renewable resources transitions. The results demonstrated 576 individual WRRFs in the CONUS could leverage HTL-based systems to reduce GHG emissions with financial savings, while simultaneously contributing to local energy and fertilizer production. Importantly, sensitivity analysis underscored the importance of increasing facility scale to expand the opportunity space for HTL-based wastewater solids management and valorization. To this end, a more complex design comprised of HTL-based hubs was proposed to increase the resources recovered from wastewater solids in the CONUS and to alleviate the economic and environmental burdens of WRRFs facing limited budget and stringent regulations.

As a high-level approximation, this study has several limitations. For example, due to the lack of detailed facility-level data (e.g., unit processes, wastewater solids characteristics, existing site-specific management costs and environmental impacts) and the variability of broader contextual factors (e.g., financial mechanisms, product prices, facility staffing requirement), this study made necessary (but conservative) assumptions. Future facility-specific studies may incorporate finer-resolution data and dynamic price modeling as technologies and markets continue to evolve. Additionally, while this study focused on the need for wastewater residual solids management at WRRFs, HTL-based systems could also incorporate other local organic wastes.^64–67^ More complex logistic analyses will be needed, but the targeted resource recovery framework established in this work can streamline the supply chain design and improve overall system efficiency, as WRRFs could still serve as centralized destinations for a wide variety of organic wastes. Such opportunities could be explored, including in rural areas with other renewable carbon resources (e.g., animal waste near ranches) to enable the financially driven deployment of HTL-based systems. However, several critical barriers must be addressed to fully realize this potential. For example, the quality of recovered products could be compromised given complex chemical interactions between different feedstocks.^68^ Compatibility between the HTL-produced biocrude and the current oil refinery infrastructure is another concern. Compared to the fossil-based crude oil, the HTL-produced biocrude has higher acidity and undesirable compositions (e.g., nitrogen-containing compounds).^69–71^ Though the impact is minimal in the current study due to the very low blending ratio between biocrude and crude oil (<0.01% in the individual facility analysis),^70^ site-specific evaluation of the existing oil refining technologies and spare processing capacity at bio-oil co-processing facilities would be needed to support biocrude production scale-up. In cases when biocrude co-processing with crude oil is not practically feasible (e.g., when the blending ratio exceeds a threshed, typical between 2% and 20% depending on the refining process),^70^ the hub deployment strategy could enable the construction of new standalone biocrude upgrading facilities by achieving economies of scale. Similar marketability challenges also apply to other recovered resources such as fertilizers and hydrochar, whose entry into the market may be constrained by issues related to product purity, regulatory acceptance, and market demand. These aspects warrant further experimental and modeling investigation as part of an integrated assessment of HTL-based systems at scale.

Ultimately, the successful deployment of HTL-based systems could potentially be driven by the financial gain of WRRFs and more stringent regulations on emerging contaminants (e.g., PFAS). Related to this, future work could incorporate policy implications such as tax credits (e.g., Clean Fuel Production Credit,^62^ PFAS destruction grants^73^) to further incentivize the implementation of HTL-based systems, and the framework established in this work could be applied to other countries and regions to help re-envision the wastewater treatment sector as an important player in sustainable waste management and circular economy transitions.

## Supplementary Material





## Notes

Detailed description of contextual parameters; hydrothermal system design and process conditions; TEA and LCA assumptions and parameters; selection of uncertainty distributions; supplemental uncertainty and sensitivity methodology details; supplemental results on the performance of HTL-based wastewater solids management systems (PDF)
supporting Excel file containing information on WRRFs, oil refineries, contextual parameters, TEA results, and LCA results (XLSX)
