# Supplementary material for: Hydrothermal-based Wastewater Solids Management for Targeted Resource Recovery and Decarbonization in the Contiguous U.S
Source: Environ Sci Technol. 2025 Sep 9;59(38):20389–400. doi: 10.1021/acs.est.5c07190 (PMC12490640; doi:10.1021/acs.est.5c07190)
Supplement: Supplementary file 1 [file es5c07190_si_001.pdf]

Supporting Information

for

Hydrothermal-based Wastewater Solids Management for Targeted Resource Recovery and Decarbonization in the Contiguous U.S.

Jianan Feng[1], Timothy J. Strathmann[2], Jeremy S. Guest[1,3,*]

[1] The Grainger College of Engineering, Department of Civil and Environmental Engineering, University of Illinois Urbana-Champaign, Urbana, Illinois 61801, United States

[2] Department of Civil and Environmental Engineering, Colorado School of Mines, Golden, Colorado 80401, United States

[3] Institute for Sustainability, Energy, and Environment, University of Illinois Urbana-Champaign, Urbana, Illinois 61801, United States

* Corresponding author: email: jsguest@illinois.edu phone: (217) 244-9247

This Supporting Information contains 32 pages, 17 figures, and 15 tables.

S1. Database

1. Water resource recovery facilities (WRRFs)

The database of U.S. WRRFs was obtained from El Abbadi *et al.*[1] Only WRRFs located within the contiguous U.S. (CONUS) were included in this study (**Figure S1**), which resulted in a total of 15,863 WRRFs, with 71 of them no less than 100 MGD, 854 at least 10 MGD but smaller than 100 MGD, and the rest smaller than 10 MGD. This dataset was constructed primarily based on the U.S. EPA's Clean Watersheds Needs Survey,[2–5] supplemented by the U.S. EPA's annual reports on biosolids production and disposal,[6] the U.S. DOE's survey of facilities with combined heat and power units,[7] and other national databases (see El Abbadi *et al.*[1] for a full list). The dataset included geospatial coordinates, treatment capacities and configurations, energy requirements, wastewater residual solids mass flow rates, and itemized greenhouse gas (GHG) emissions for WRRFs across the CONUS. The GHG emissions were categorized into scope 1 emissions from direct onsite processes (e.g., methane, nitrous oxide, fossil-derived carbon dioxide), scope 2 emissions from upstream energy generation, and scope 3 emissions from upstream chemical production and downstream residual solids disposal. All emissions were estimated using an emission factor-based approach coupled with Monte Carlo simulations. However, the dataset did not contain detailed unit processes for onsite wastewater residual solids treatment. As a result, emissions from onsite solids treatment (e.g., fugitive emissions, energy-related emissions) were not separately identified and are instead aggregated under facility-level emissions. The full dataset is provided in the **supporting Excel file,** and further methodological details can be found in El Abbadi *et al.*[1]

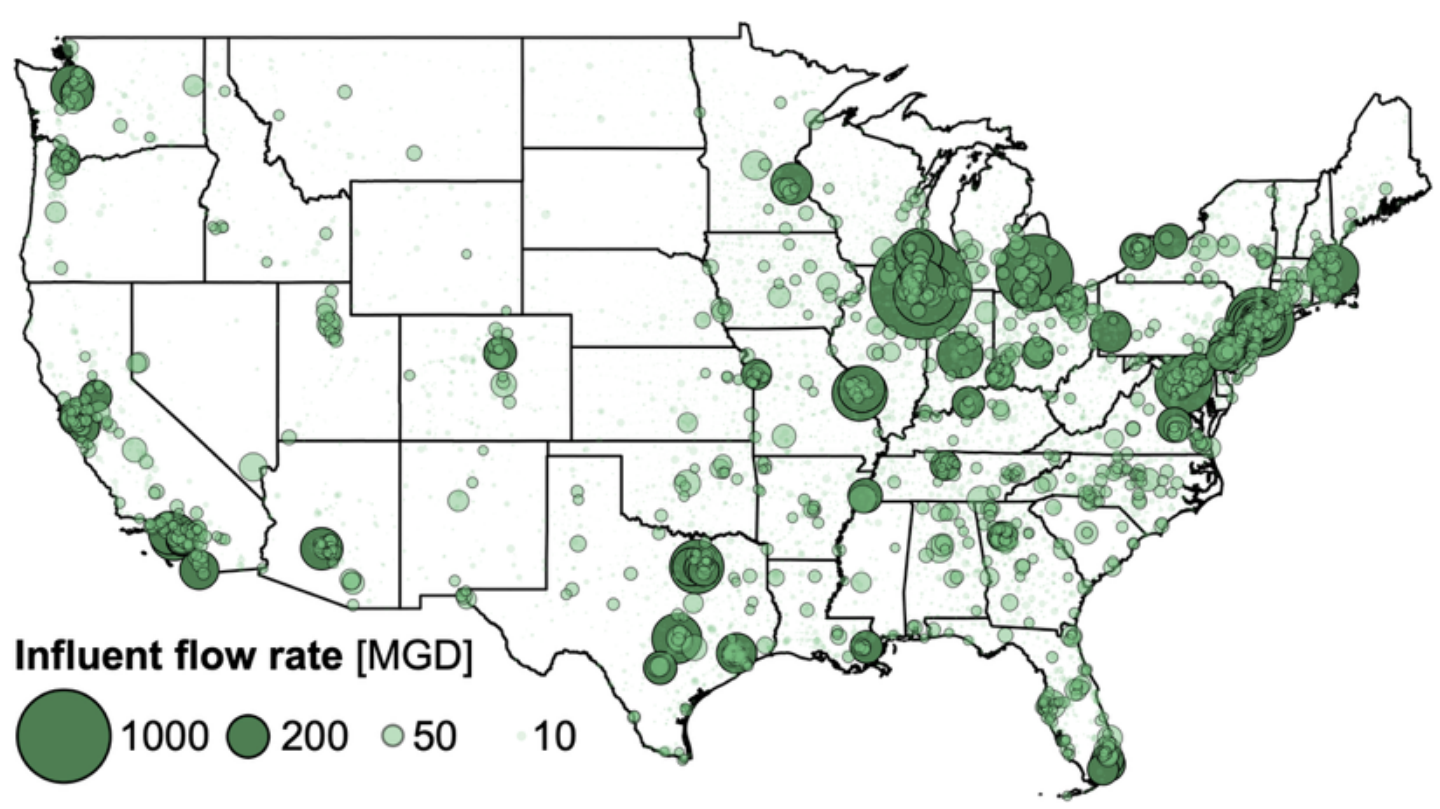

Figure S1. The distribution of water resource recovery facilities (WRRFs) across the contiguous U.S. Each circle represents one WRRF, with the color and the size indicating the design capacity of the WRRF. The legends from left to right represent influent flow rate of 1,000, 200, 50, and 10 MGD.

2. Oil refineries

The database of U.S. oil refineries was obtained from 2024 U.S. Energy Information Administration (EIA) refinery capacity report.[8] Only oil refineries located within the CONUS and with a non-zero capacity were included in this study (**Figure S2**). The oil refinery data were categorized based on the Petroleum Administration for Defense Districts (PADDs) into five groups: PADD 1 (East Coast), PADD 2 (Midwest), PADD 3 (Gulf Coast), PADD 4 (Rocky Mountain), and PADD 5 (West Coast). Detailed data on oil refineries were provided in the **supporting Excel file**.

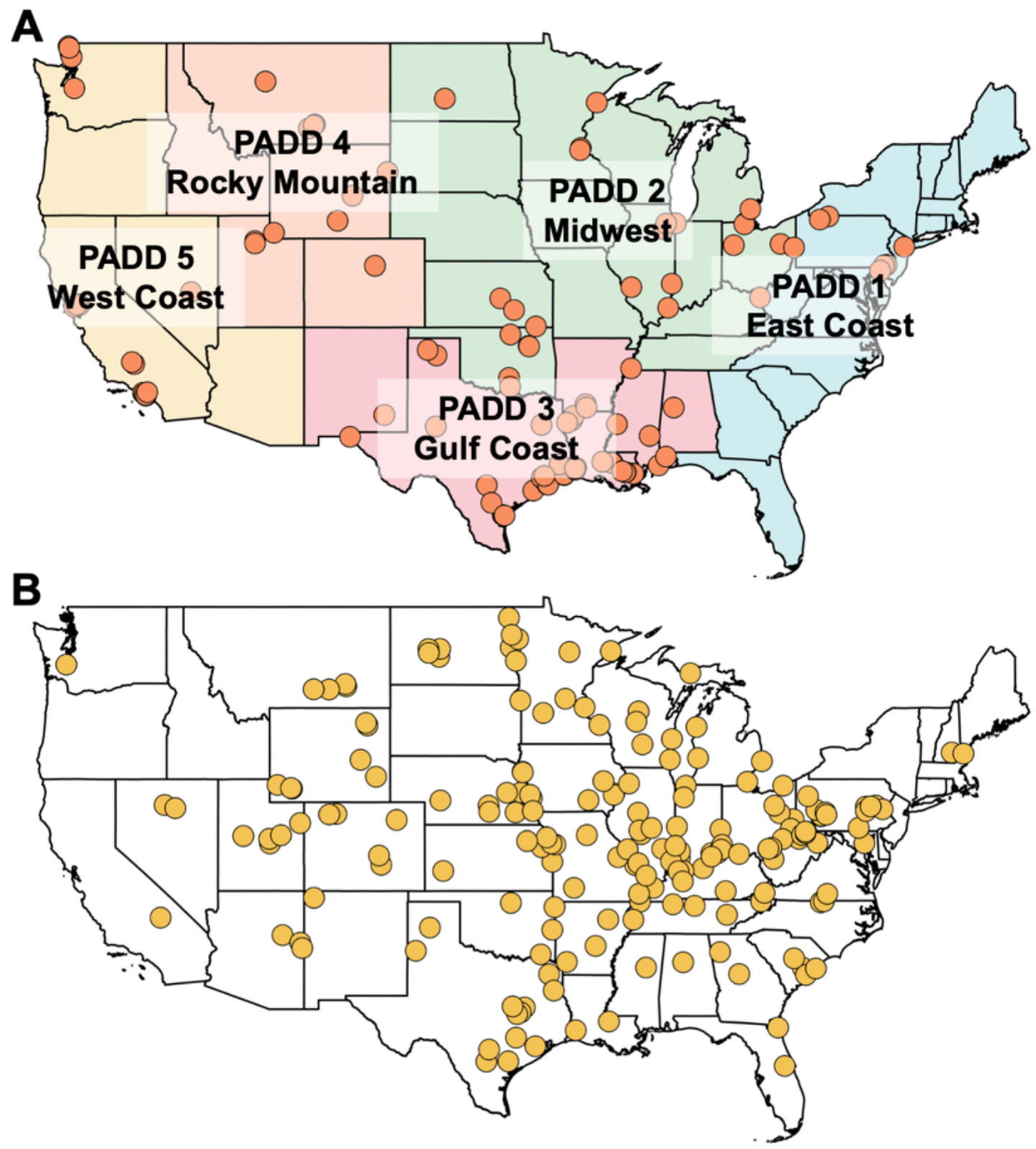

Figure S2. The distribution of (A) oil refineries and (B) coal-based power plants across the contiguous U.S. Each circle represents one facility.

3. Nitrogen and phosphorus fertilizer demands

County-level nitrogen and phosphorus fertilizer demand data were obtained from the U.S. Geological Survey.[9] Only counties located within the CONUS were included in this study (**Figure S3**). The fertilizer data include both farm fertilizer and non-farm fertilizer and their sums were considered. The form of nitrogen fertilizers was based on Cao *et al.*[10] The results were categorized into seven regions: Northwest, Southwest, Northern Great Plains, Southern Great Plains, Midwest, Southeast, and Northeast. The regional ranking of major nitrogen fertilizers (i.e., anhydrous ammonia, urea, and urea ammonium nitrate [UAN]) is included in **Table S1**. The form of phosphorus fertilizers was based on U.S. Department of Agriculture[11] and Cao *et al.*,[10] with diammonium phosphate (DAP) as the dominant form regardless on locations. Detailed data on nitrogen and phosphorus fertilizer data were provided in the **supporting Excel file**.

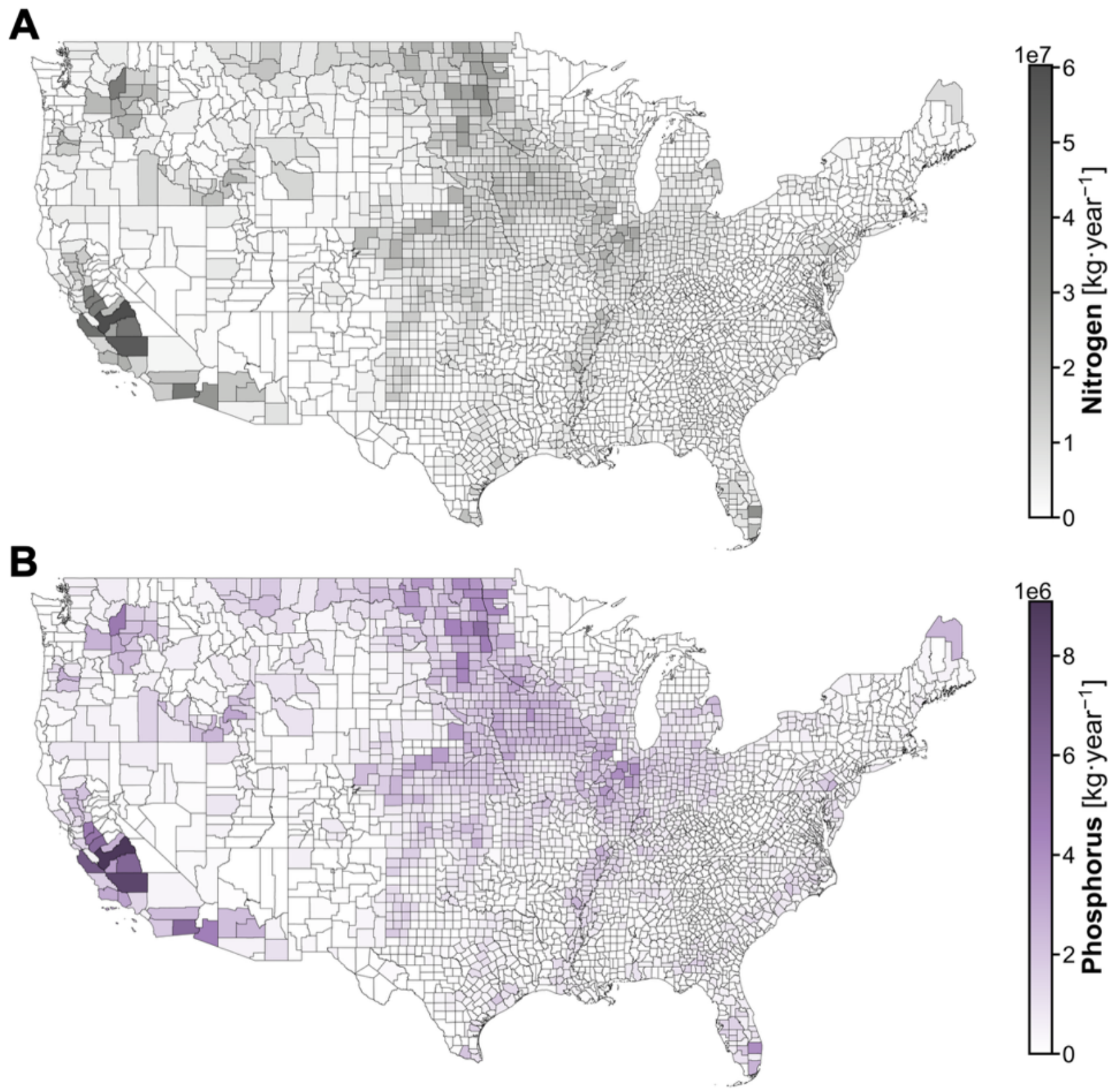

Figure S3. County-level (A) nitrogen and (B) phosphorus fertilizer demands across the contiguous U.S.

Table S1. The regional ranking of major nitrogen fertilizers.

Region	Nitrogen fertilizer ranking		
	**1st**	**2nd**	**3rd**
Northwest	urea	UAN	anhydrous ammonia
Southwest	UAN	anhydrous ammonia	urea
Northern Great Plains	urea	anhydrous ammonia	UAN
Southern Great Plains	UAN	urea	anhydrous ammonia
Midwest	anhydrous ammonia	UAN	urea
Southeast	UAN	urea	ammonium sulfate
Northeast	UAN	urea	ammonium sulfate

S2. Contextual parameters at baseline

1. Electricity price and carbon intensity

The different energy profiles in different regions lead to varied electricity price and carbon intensity (CI). In this study, the 2022 state-level electricity price[12] was considered (**Figure S4A**) and the electricity CI was calculated based on the 2022 projected grid composition[13] for 134 modeling regions in the contiguous U.S. (**Figure S4B**), following the same method described in El Abbadi *et al.*[1] Detailed data on electricity price and CI were provided in the **supporting Excel file**.

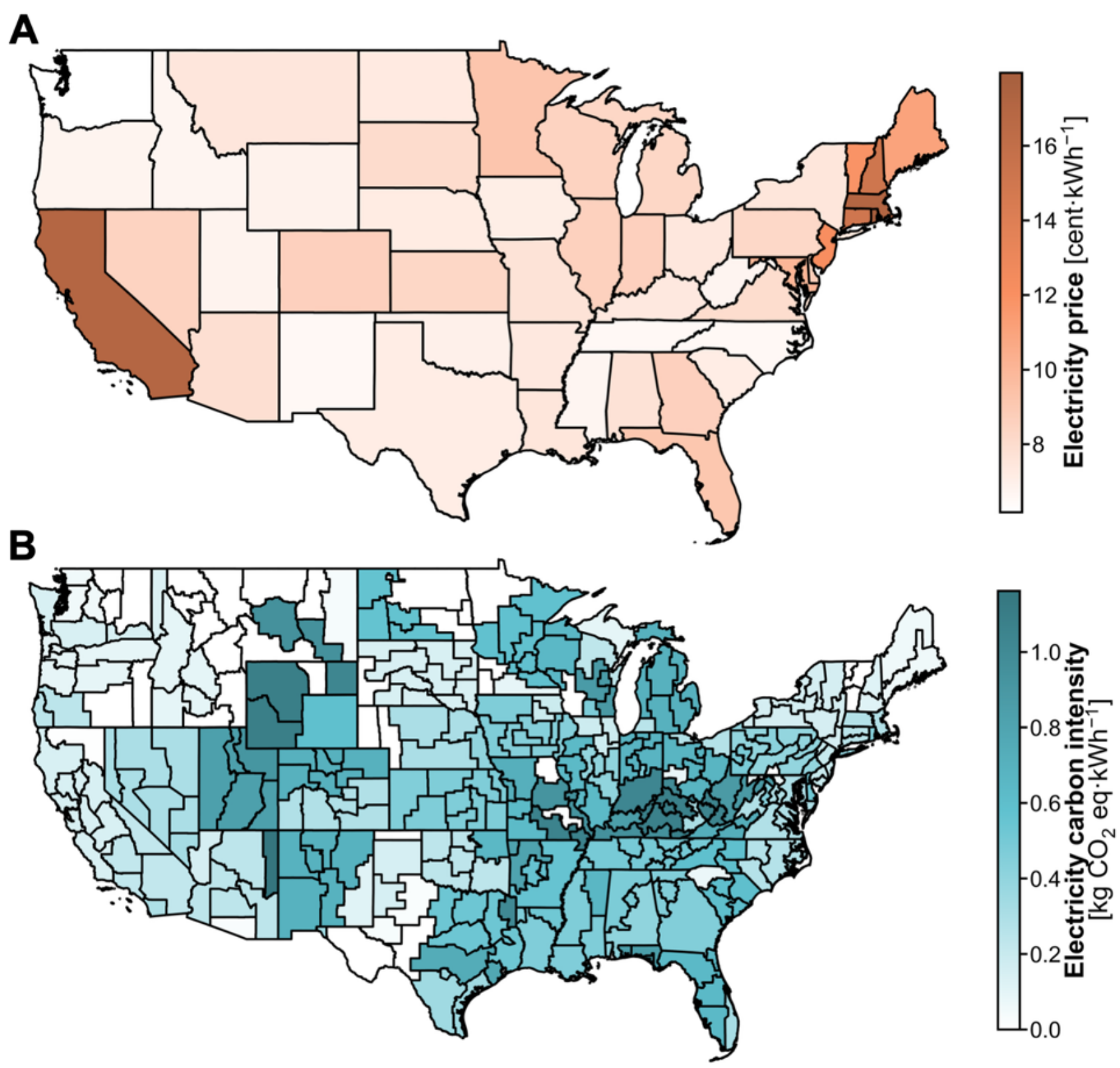

Figure S4. (A) State-level electricity price and (B) regional-level electricity carbon intensity in the contiguous U.S. in 2022.

2. Crude oil and fertilizer prices

The first purchasing price of crude oil was obtained from U.S. Energy Information Administration (**Supporting Excel document**).[14] It was assumed the oil refinery purchased biocrude at WRRFs, therefore the price of crude oil was based on the WRRFs location. For states with no available crude oil price data, the PADD average data was applied. The price of crude oil was converted to the price of biocrude based on their higher heating values (HHV). The baseline HHV were 44.5 $MJ{\cdot}kg^{-1}$ for crude oil and 35 $MJ{\cdot}kg^{-1}$ for HTL-produced biocrude, while the HHV of HTL-produced biocrude was additionally calculated using the Dulong equation (below) in the uncertainty analysis:

$$HHV\ [MJ{\cdot}kg^{-1}] = 0.338 \times C\% + 1.428 \times (H\% - 0.125 \times O\%)$$

where C%, H%, and O% represent dry weight% of carbon, hydrogen, and oxygen, respectively.

The 2022 fertilizer price was obtained from U.S. Department of Agriculture (**Table S2**).[15] Data were only available for 6 states and the average of minimum and maximum values was used as the baseline. For states with no available data, the average of minimum and maximum values from states with available data was used.

Table S2. 2022 fertilizer price [$·tonne^{-1}] in the contiguous U.S.

State	DAP			anhydrous ammonia			urea			UAN[a]		
	min	average	max	min	average	max	min	average	max	min	average	max
AL	648	1,070	1,500	-	-	-	634	937	1,240	617	777	937
IA	915	1,100	1,290	1,170	1,530	1,880	717	956	1,190	586	726	865
IL	882	1,060	1,240	1,200	1,560	1,930	772	965	1,160	601	774	948
NC	671	1,100	1,520	-	-	-	493	942	1,390	386	667	948
OK	882	1,130	1,370	1,210	1,450	1,680	772	973	1,170	457	670	882
SC	904	1,110	1,310	-	-	-	717	987	1,260	496	648	799
others	648	1,090	1,520	1,170	1,550	1,930	493	942	1,390	386	667	948

[a] Including UAN-28, UAN-30, and UAN-32.

3. Labor wage

The 2022 county-level labor wage data for local government-paid positions were obtained from the U.S. Bureau of Labor Statistics.[16] Quotients between county-level data and the U.S. average value were calculated (**Figure S5**) to adjust the labor cost in the techno-economic analysis (TEA). Detailed data on labor wage were provided in the **supporting Excel file**.

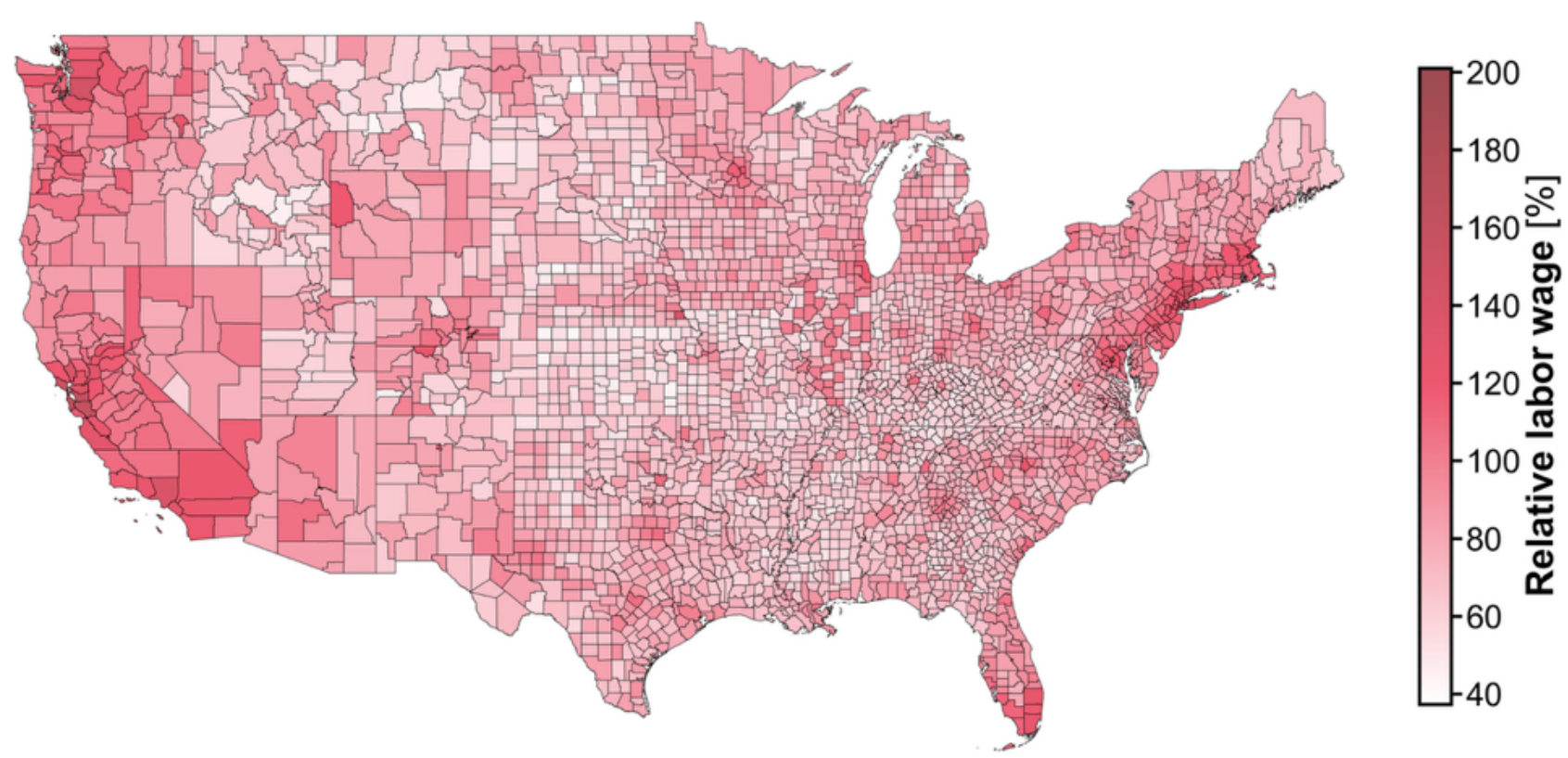

Figure S5. County-level labor wage in the contiguous U.S. in 2022.

4. Corporate income tax

The corporate income tax is composed of federal corporate income tax (21% in 2022) and state-level income tax (and/or gross receipts tax).[17] The detailed tax brackets are available in the Python code.[18] Top marginal tax rates are shown in **Figure S6**.

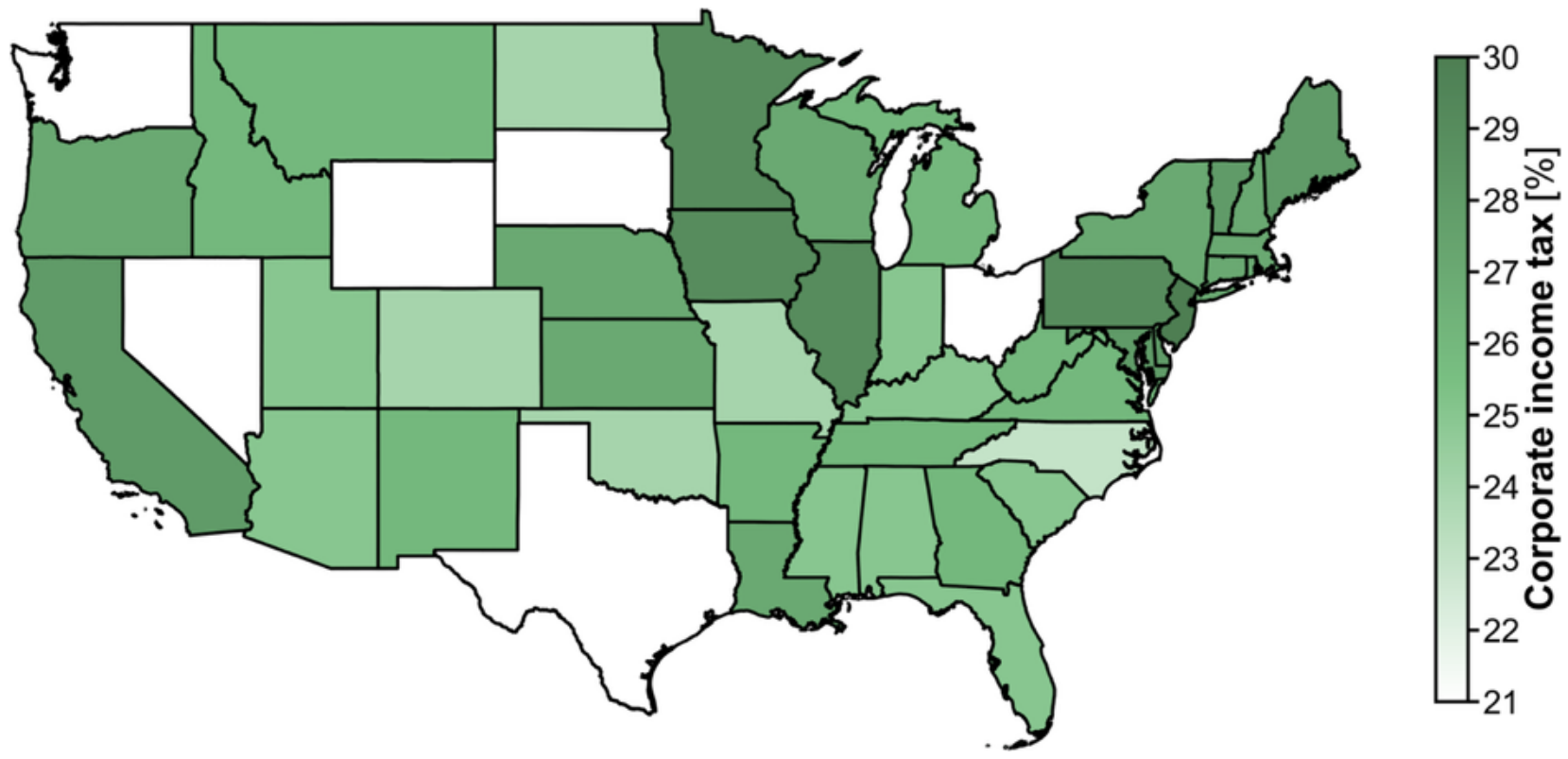

Figure S6. Top marginal state-level corporate income tax (and/or gross receipts tax) in the contiguous U.S. in 2022.

S3. HTL-based wastewater solids management model

1. System description

We used two open-source Python packages—QSDsan (using the 'pfas' branch as updated as of July 31, 2025)[19] and BioSTEAM (using the 'qsdsan' branch as updated as of June 18, 2025)[20]—for the design and analysis of the hydrothermal liquefaction (HTL)-based wastewater solids management system. The major units/processes in the system included the HTL for wastewater solids valorization, hydrothermal gasification for fuel gas production, multiple nutrient recovery units, a heat exchanger network for heat integration, a combined heat and power unit for heating and electricity generation, a chilled water package and a cooling tower for cooling. All units were coded using Python 3.9 and could be publicly accessed online.[18] The system design primarily followed our previous work,[21] but the following changes have been made.

1. All WRRFs in the CONUS were assumed to have already implemented wastewater solids dewatering to a moisture content of 80% and, therefore, no dewatering unit was included. The impact of this assumption was minimal as demonstrated in the sensitivity analyses conducted by Feng *et al.*[21]
2. Different ash content (on a dry weight [dw] basis) and biochemical compositions (lipid, protein, and carbohydrate content on an ash-free dry weight [afdw] basis) of wastewater solids were assumed for WRRFs without solids digestion, with anaerobic digestion, with aerobic digestion, and with both anaerobic digestion and aerobic digestion (**Table S3**).
3. The minimum approaching temperature of the heat exchanger network was assumed to be the default value in BioSTEAM (5°C) rather than being set manually.
4. To further reduce GHG emissions associated, an amine absorption unit was modeled to capture CO_2 from the flue gas released by the combined heat and power unit. Captured CO_2 was assumed to be used for the production of nitrogen fertilizers.
5. Biocrude was assumed to be transported to oil refineries (instead of onsite refinement). Hydrochar was assumed to be transported to coal-based power plants for co-firing. Nitrogen was assumed to be recovered as anhydrous ammonia, urea, or UAN based on the location of WRRFs. Phosphorus was assumed to be recovered as DAP. Detailed descriptions of newly added units/processes are included in the following subsections.

Table S3. Baseline characteristics of wastewater solids from different solid treatment methods.

Description	Count	Ash [dw%]	Lipid [afdw%]	Protein [afdw%]	Carbohydrate [afdw%]	Ref.
no digestion	181	23.1%	20.6%	45.6%	1 − lipid − protein	[22]
anaerobic digestion	2,698	41.4%	19.3%	51.0%		[22]
aerobic digestion	12,539	43.6%	19.3%	51.0%		[a]
both anaerobic and aerobic digestion	437	43.6%	19.3%	51.0%		[b]

[a] Assuming aerobic digestion results in a higher ash content but the same biochemical compositions as anaerobic digestion.

[b] If a WRRF has both anaerobic digestion and aerobic digestion, assuming ash content and biochemical compositions follow the case of aerobic digestion to be conservative (due to a higher ash content).

2. Biocrude and hydrochar transportation

HTL-produced biocrude and hydrochar were transported to nearby oil refineries and coal-based power plants, respectively. Therefore, hydrotreating, hydrocracking, and their associated units (e.g., flash, distillation columns) for onsite biocrude upgrading were removed. WRRFs and fossil energy facilities were matched based on their near transportation distances using the *sjoin_nearest* function in the Python package—*GeoPandas* (version 0.13.0).[23] For matched facilities, the *distance_matrix* function in the Python Client for Google Maps Services (version 4.10.0) was used for distance calculation under the 'driving' mode.[24] Eight WRRFs that cannot be reached by driving (e.g., on an isolated island) were excluded. Removed WRRFs were no larger than 3 MGD, and the impact was minimal.

3. DAP recovery

DAP is produced through phosphorus acid extraction followed by precipitation. Specifically, 5% H_2SO_4 solution was used to extract phosphorus from HTL-produced hydrochar at a baseline acid-to-solids ratio of 7 $mL \cdot g^{-1}$. Then, stripped NH_3 (see **Section 3.4**) was added into the extractant to allow the formation of DAP. The mixed solution was then transferred to batch crystallizers to allow the formation of DAP crystal, which was then separated from the solution using a pressure filter.

4. Anhydrous ammonia recovery

Anhydrous ammonia is produced through air stripping and drying. The air stripping was done using water steam at 117°C generated from a boiler. The stripping process was modeled as an adiabatic multi-stage distillation column capable of vapor-liquid equilibrium but without a reboiler or condenser. The diameter of the column was based on tray separation and flooding velocity and the purchase costs were based on correlations compiled by Seider *et al.*[25] Then, part of the stripped ammonia was sent to the DAP synthesizer and the rest being dried through passing multiple molecular sieves. The anhydrous ammonia was converted to liquid form by compressing it to 2 MPa and cooling it to 25°C.

5. Urea recovery

As a key reactant for urea synthesis, CO_2 was captured from the flue gas of the CHP unit using 30 wt% aqueous monoethanolamine (MEA). Capital costs of the CO_2 capture unit were based on Devkota *et al.*[26] Urea was then synthesized through reacting CO_2 and dried ammonia, with excess amount of dried ammonia (at a baseline anhydrous ammonia to CO_2 ratio of 3.5:1) to ensure the high conversion efficiency of CO_2 to urea (at a baseline efficiency of 0.8). The unreacted anhydrous ammonia and CO_2 were recirculated. At the same time, a loss factor (at a baseline value of 0.02) was assumed to account for loss of reactants during reactions. Capital costs of the urea synthesizer were based on Devkota *et al.*[26] Electricity and heat demands were based on Palys *et al.*[27]

6. UAN recovery

UAN was produced by further mixing produced urea with ammonium nitrate, which was produced by mixing produced dried ammonia with nitric acid solution. The final nitrogen content in UAN is 30 wt%.

S4. Techno-economic analysis (TEA) and life cycle assessment (LCA)

1. General assumptions

TEA and LCA followed the methodology from Feng *et al.*[21] The system boundary was expanded to include the transportation of biocrude to oil refineries and the transportation of wastewater solids to HTL-based hubs, if relevant. Construction was excluded from LCA per the guideline of the Renewable Fuel Standard.[28] TEA and LCA focused on financial savings and GHG reductions as displacing incumbent wastewater solids management methods with HTL-based systems. Avoided management costs[29] and GHG emissions[1] were calculated based on the percentage share of different management methods (**Table S4**). It should be noted CO_2 emissions from incineration was assumed to be biogenic, thus not contributing to GHG emissions. For landfilling and land application, only fugitive emissions were considered as emissions from wastewater solids hauling were relatively negligible (**Figure S7**); this observation assumes (1) hauled wastewater solids have a moisture content of 80%, (2) the median and average one-way distance for land application are 8 km and 13 km, respectively, based on data from representative WRRFs,[30] and (3) hauling distance for landfilling can be 1 to 2 times as that of land application but was not considered due to the lack of detailed data (hauling related CI can be found in **Table S6**).

Table S4. Costs and GHG emissions of conventional wastewater solids management methods.

**Management practice**	**Cost** [$·tonne^{-1}]	**GHG emission** [kg CO_2 eq·tonne^{-1}]
landfilling	413	168
land application	606	210
incineration	441	0

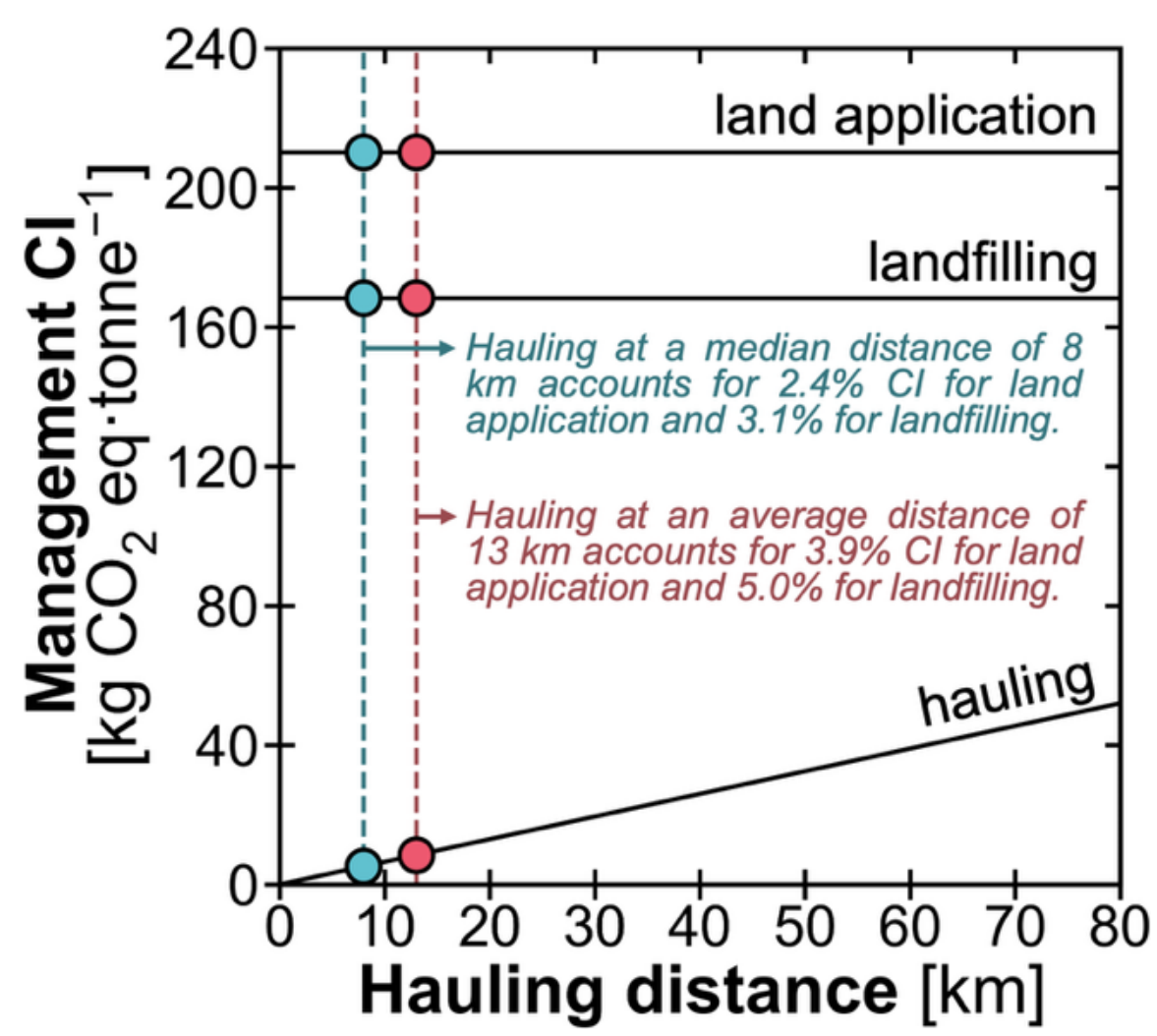

Figure S7. GHG emissions of wastewater solids hauling compared to fugitive GHG emissions from landfilling and land application at different hauling distances.

2. TEA

The estimation of capital expenditures (CAPEX) and operation and maintenance expenditures (OPEX) were automated using existing algorithms in QSDsan based on design of each individual unit included in the system. A list of assumptions for TEA were included in **Table S5**. Compared

to the previous work,[21] we adjusted the income tax and labor cost based on geospatial locations and system sizes, respectively. Specifically, income tax was calculated as the sum of the federal corporate income tax (21%) and state corporate income tax (and/or gross receipts tax for states levying gross receipts tax). The state corporate income tax varied from 0% to 11.5% and can be determined based on location and income brackets.[31] If no state was specified, an U.S. average state income tax of 6.5% was used.[32] The labor cost in this study was divided into managerial (plant manager, plant engineer, maintenance supervisor, and lab manager) and other employees (e.g., operators, technicians, secretaries) and was converted to 2022$ using the Employment Cost Index.[33] The managerial labor cost was kept constant at 0.43 MM $·yr^{-1},[34] with other labor costs linearly scaled based on the mass flow rate of wastewater solids using the equation from Leow *et al.*[35] (assuming the baseline was 0.61 MM $·yr^{-1} for a system managing 100 tonne wastewater solids per day[34]). After calculating the CAPEX and the OPEX, the discounted cash flow rate of return analysis was performed to calculate financial savings.

Table S5. TEA baseline assumptions for HTL-based waste solids management systems.

Parameters	Values
internal rate of return	3%
project lifetime	30 years
income tax	21% (federal) + X% (state)
yearly operating days	365
construction schedule	3 years (8% 1st year, 60% 2nd year, 32% 3rd year)
startup month	6
startup fixed operating cost fraction	1
startup sales fraction	0.5
startup volatile operating cost fraction	0.75
working capital over fixed capital investment (FCI)	5%
finance interest	3%
finance years	10
finance fraction	0.6
warehouse	4% of installed equipment cost
site development	9% of inside battery limits (ISBL)
additional piping	4.5% of ISBL
proratable costs	10% of total direct cost (TDC)
field expenses	10% of TDC
home office and construction	20% of TDC
project Contingency	10% of TDC
other indirect costs	10% of TDC
labor cost [$·yr^{-1}]	$4.3\times10^5 + 6.1\times10^5 \times(\text{scale}/100)$[a]
labor burden	90%
property insurance and taxes	0.7% of FCI
maintenance	3% of total indirect cost (TIC)
steam power depreciation	MACRS20[b]

[a] scale: daily wastewater solids management amount [tonne·day^{-1}]

[b] MACRS: modified accelerated cost recovery system

3. LCA

The environmental impacts of HTL-based wastewater solids management system were estimated based on the material requirements and emissions during the operation stage of the facility. LCA was conducted with a focus on the carbon intensity (CI; a.k.a. global warming potential, GWP) to characterize the system-level GHG emissions. Unless otherwise noted, we used Ecoinvent v3.8 database (cutoff model)[36] for the life cycle inventory data and the U.S. EPA's Tool for the Reduction and Assessment of Chemicals and Other Environmental Impacts (TRACI)[37] for characterization factors (**Table S6**).

Table S6. LCA inventory data for raw materials and energy sources.

Raw Materials, Ancillary Inputs, Unit Processes, Energy Sources	Unit	CI [kg CO_2-eq]
CH_4	kg	29.8[a]
N_2O	kg	273[a]
CHG catalyst	kg	471
5% H_2SO_4	kg	0.00553
deionized water	kg	0.000420
NaOH	kg	1.25
natural gas (kg)	kg	3.47
crude oil[b]	kg	0.223
hard coal[c]	kg	0.170
ash disposal	kg	0.00827
MEA	kg	3.09
HNO3	kg	2.32
DAP	kg	1.46
anhydrous ammonia	kg	2.48
urea	kg	1.25
UAN	kg	1.68
transportation/hauling	tonne·km	0.130
electricity	kWh	[d]

[a] From the Intergovernmental Panel on Climate Change (IPCC).[38]

[b] Assuming HTL-produced biocrude displacing crude oil based on the energy equivalence, with the baseline HHV of crude oil being 44.5 MJ·kg^{-1} and the HHV of biocrude calculated using the Dulong equation.

[c] Assuming HTL-produced hydrochar displacing hard coal based on the energy equivalence, with the baseline HHV of hard coal being 27.9 MJ·kg^{-1} and the HHV of hydrochar calculated using the Dulong equation.

[d] Included in the **supporting Excel document**.

Table S7. Itemized lifetime GHG emissions of HTL-based systems in Feng *et al.*[21]

systems	**itemized lifetime GHG emissions** [k-tonne CO_2 eq]			construction share
	construction	material	utility	
system A (**Figure S3**)	5.1	−105	459	1.4%
system B (**Figure S4**)	5.0	−159	420	1.9%
system C (**Figure S5**)	4.9	-84	534	1.1%

S5. Uncertainty and sensitivity analyses

1. Facility-level uncertainty

For WRRFs with both baseline decarbonization amount and baseline saving greater than 0, we performed uncertainty analysis to reflect possible ranges of the parameters. We used a tiered system with selection criteria, consistent with the approach described in Feng *et al.*,[21] to determine the types and ranges of uncertainty distributions.

- Criterion 1. For parameters where the distributions can be found in literature, we used the same distributions as the literature.
- Criterion 2. For parameters where only the ranges can be found in literature, we chose a uniform distribution with the minimum and maximum limits as the lower and upper bounds of the distribution and the average value of the minimum and maximum limits as the baseline.
- Criterion 3. For parameters where only a single value can be found in the literature and is based on empirical formulae, this value was assigned as the baseline and uniform distributions were set from 90% to 110% of the baseline value.
- Criterion 4. For parameters with no known distribution in the literature:
  - Criterion 4.1. If more than 20 data points were found in the literature, we selected triangular distributions and used 5th, 50th, and 95th percentiles from the literature values as the minimum, most probable, and maximum values.
  - Criterion 4.2. If only 5 to 20 data points were found in the literature, we selected triangular distributions and used minimum, average, and maximum of the literature value as the minimum, most probable, and maximum values.
  - Criterion 4.3. If less than 5 data points can be found in the literature, we selected uniform distributions and used 80%, 100%, and 120% of the average value as lower bound, baseline, and upper bounds.

A complete list of distributions for each parameter used in the model can be found below (**Table S8**).

Table S8. List of parameters included in the uncertainty analyses.

Parameters		Unit	Criterion	Distribution	Lower	Baseline	Upper	Std. Dev.
Waste solids characteristics								
solids wet density		$kg\cdot m^{-3}$	#2	uniform	1,000	1,040	1,080	
undigested solids	ash ratio (dw)[a]	-	#4.2	triangular	0.174	0.231	0.308	
	lipid ratio (afdw)[a]	-	#4.2	triangular	0.080	0.206	0.308	
	protein ratio (afdw)[a]	-	#4.2	triangular	0.380	0.456	0.485	
aerobically digested solids	ash ratio (dw)[a]	-	#4.3	uniform	0.349	0.436	0.523	

	lipid ratio (afdw)[a]	-	#4.3	uniform	0.154	0.193	0.232	
	protein ratio (afdw)[a]	-	#4.3	uniform	0.408	0.510	0.612	
	ash ratio (dw)[a]	-	#4.3	uniform	0.331	0.414	0.497	
anaerobically digested solids	lipid ratio (afdw)[a]	-	#4.3	uniform	0.154	0.193	0.232	
	protein ratio (afdw)[a]	-	#4.3	uniform	0.408	0.510	0.612	
lipid carbon content		-	#3	uniform	0.675	0.75	0.825	
protein carbon content		-	#3	uniform	0.491	0.545	0.600	
carbohydrate carbon content		-	#3	uniform	0.36	0.4	0.44	
lipid hydrogen content		-	#3	uniform	0.113	0.125	0.138	
protein hydrogen content		-	#3	uniform	0.0614	0.0682	0.075	
carbohydrate hydrogen content		-	#3	uniform	0.06	0.0667	0.0733	
protein to nitrogen ratio		-	#3	uniform	0.143	0.159	0.175	
nitrogen to phosphorus ratio		-	#4.2	triangular	0.194	0.393	0.556	
HTL								
enforced heat transfer coefficient of HTL heat exchanger		$kW·m^{-2}·K^{-1}$	#2	triangular	0.0170	0.0795	0.0852	
lipid to biocrude[b]		-	#1	triangular	0.692	0.846	1	
protein to biocrude[b]		-	#1	normal		0.445		0.030
carbohydrate to biocrude[b]		-	#1	normal		0.205		0.050
protein to off-gas[b]		-	#1	normal		0.074		0.020
carbohydrate to off-gas[b]		-	#1	normal		0.418		0.030
biocrude carbon slope[b]		-	#1	normal		−8.37		0.939
biocrude carbon intercept[b]		-	#1	normal		68.6		0.367
biocrude nitrogen slope[b]		-	#1	normal		0.133		0.005
biocrude hydrogen slope[b]		-	#1	normal		−2.61		0.352
biocrude hydrogen intercept[b]		-	#1	normal		8.20		0.138
HTL aqueous carbon slope[b]		-	#1	normal		478		18.88
hydrochar carbon slope[b]		-	#1	normal		1.750		0.122
hydrochar hydrogen slope		-	#4.3	uniform	0.113	0.141	0.169	
HTL aqueous TOC to TC ratio		-	#1	triangular	0.715	0.764	0.813	
biocrude moisture content		-	#4.2	triangular	0.035	0.0631	0.102	
biocrude wet density		$kg·m^{-3}$	#4.2	triangular	950	983	1,010	

crude oil density	$kg\cdot m^{-3}$	#2	uniform	800	850	900
crude oil higher heating value (HHV)	$MJ\cdot kg^{-1}$	#2	uniform	42	44.5	47
hard coal HHV	$MJ\cdot kg^{-1}$	#3	uniform	25.1	27.9	30.7
CHG						
weight hourly space velocity	$kg\cdot hr^{-1}\cdot kg\ catalyst^{-1}$	#4.2	triangular	2.86	3.56	3.99
catalyst lifetime	hr	#1	triangular	3,960	7,920	15,800
gas carbon to total carbon ratio	-	#4.2	triangular	0.189	0.598	0.780
effluent pH	-	#2	uniform	7.91	8.16	8.41
Acid extraction & DAP synthesis						
acid to hydrochar	$mL\cdot g^{-1}$	#2	uniform	4	7	10
phosphorus extraction ratio	-	#2	uniform	0.7	0.8	0.9
phosphorus to DAP	-	#4.2	triangular	0.7	0.828	0.95
CO_2 capture						
CO_2 recovery	-	#3	uniform	0.81	0.9	0.99
MEA to CO_2	-	#3	uniform	1.35	1.5	1.65
Urea and UAN syntheses						
NH_3 to CO_2	-	#4.3	uniform	2.8	3.5	4.2
CO_2 to urea	-	#4.3	uniform	0.64	0.8	0.96
loss ratio	-	#4.3	uniform	0.016	0.02	0.024
TEA						
internal rate of return	-	#1	triangular	0%	3%	5%
HTL CAPEX factor	-	#1	triangular	0.6	1.0	1.4
CHG CAPEX factor	-	#1	triangular	0.6	1.0	1.4
CHP unit CAPEX	$\$\cdot kW^{-1}$	#4.3	uniform	980	1,230	1,470
5% H_2SO_4 price	$\$\cdot kg^{-1}$	#3	uniform	0.00596	0.00662	0.00729
NaOH price	$\$\cdot kg^{-1}$	#3	uniform	0.568	0.631	0.695
CHG catalyst (7.8% Ru/C) price	$\$\cdot kg^{-1}$	#1	triangular	85.3	171	341
deionized water price	$\$\cdot kg^{-1}$	#3	uniform	0.000477	0.000530	0.000583
natural gas price	$\$\cdot kg^{-1}$	#3	uniform	0.196	0.218	0.240
$(NH_4)_2SO_4$ price	$\$\cdot kg^{-1}$	#4.2	triangular	0.22	0.283	0.32
MEA price	$\$\cdot kg^{-1}$	#4.2	triangular	1.93	2.13	2.31

HNO_3 price	$\$\cdot kg^{-1}$	#4.2	triangular	0.43	0.497	0.53
crude oil price[a]	$\$\cdot kg^{-1}$	#4.2	triangular	see **supporting Excel document**		
DAP price[a]	$\$\cdot kg^{-1}$	#4.2	triangular	see **Table S2**		
anhydrous ammonia price[a]	$\$\cdot kg^{-1}$	#4.2	triangular	see **Table S2**		
urea price[a]	$\$\cdot kg^{-1}$	#4.2	triangular	see **Table S2**		
UAN price[a]	$\$\cdot kg^{-1}$	#4.2	triangular	see **Table S2**		
hydrochar price	$\$\cdot kg^{-1}$	#2	uniform	0	0.0536	0.107
ash disposal cost	$\$\cdot kg^{-1}$	#3	uniform	0.0453	0.0503	0.0554
electricity price	$\$\cdot kWh^{-1}$	#3	uniform	0.9*X	1*X	1.1*X
labor cost adjustment	-	#3	uniform	0.9*X	1*X	1.1*X
biocrude transportation distance	km	#3	uniform	1*X	1*X	1.1*X
hydrochar transportation distance	km	#3	uniform	1*X	1*X	1.1*X
sludge transportation distance	km	#3	uniform	1*X	1*X	1.1*X
fixed transportation price (biocrude)	$\$\cdot m^{-3}$	#3	uniform	6.84	7.60	8.37
variable transportation price (biocrude)	$\$\cdot m^{-3}\cdot km^{-1}$	#3	uniform	0.0845	0.0939	0.103
fixed transportation price (hydrochar)	$\$\cdot tonne^{-1}$	#4.3	uniform	4.25	5.32	6.38
variable transportation price (hydrochar)	$\$\cdot tonne^{-1}\cdot km^{-1}$	#4.3	uniform	0.0417	0.0522	0.0626
fixed transportation price (solids)	$\$\cdot m^{-3}$	#3	uniform	4.98	5.53	6.08
variable transportation price (solids)	$\$\cdot m^{-3}\cdot km^{-1}$	#3	uniform	0.0488	0.0543	0.0597
LCA						
impact indicators for GWP listed in **Table S6**		#3	uniform	0.9*X	1*X	1.1*X

[a] Assume uniform distributions with ± 20% uncertainty around baseline values for reconstituted WRRFs and for hub analyses involving wastewater solids co-processing across multiple WRRFs.

[b] Used in a multiphase additivity model.[35,39]

2. Multi-facility uncertainty aggregation with Gaussian copula

Due to shared parameters (**Table S8**) within HTL-based models for WRRFs, Gaussian copula was used to model the dependence (i.e., inter-correlation) when aggregating individual WRRF results. One key benefit of the Gaussian copula is it has no tail dependence, meaning extreme results (outliers) from one WRRF are not necessarily linked to extreme results in other WRRFs. The analysis was conducted through the following steps:

1. Generating a n-dimension matrix (N) of standard normal distributions (N_i).

$$N = [N_1 \quad \dots \quad N_n]$$

2. Defining a n-dimension correlation matrix (A), where $\rho_{i,j}$ represents the correlation coefficient between results from the ith and the jth WRRFs. All $\rho_{i,j}$ values were assumed to be equal. The effect of different $\rho_{i,j}$ values was tested and the results showed while the median aggregate outcomes remained stable, the uncertainty bounds narrowed substantially as $\rho_{i,j}$ decreased (**Table S13**). This reflected the effect of unrealistic independence assumptions at low coefficients, which artificially smoothed aggregate outputs and underestimated true uncertainties. Therefore, a $\rho_{i,j}$ of 0.8 was selected to represent a strong but not extreme positive correlation across WRRFs, while preserving a reasonable and realistic uncertainty range.

$$A = \begin{bmatrix} 1 & \cdots & \rho_{1,n} \\ \vdots & \ddots & \vdots \\ \rho_{n,1} & \cdots & 1 \end{bmatrix}$$

3. Computing the upper-triangular Cholesky matrix (L*) to introduce dependency. L denotes the lower-triangular Cholesky matrix.

$$A = LL^*$$

4. Generating dependent samples (D), converting D into to a uniform distribution (i.e., quantile; Q) using the cumulative distribution function (CDF), and mapping the dependent samples (S') to the original samples (S).

$$D = NL^T$$
$$Q = CDF(D)$$
$$S' = Q(S)$$

5. Aggregating samples to get the final uncertainty distribution (R).

$$R = \sum S'$$

3. Agglomerative Hierarchical Clustering

Due to the high number of WRRFs (i.e., 15,855), Agglomerative Hierarchical Clustering (i.e., an unsupervised machine learning algorithm) was used to group WRRFs into 1,000 clusters to improve the simulation efficiency for the sensitivity analysis. The agglomerative hierarchical clustering was conducted through the following steps:

1. Selecting the following parameters for clustering: wastewater solids mass flow rate, solid digestion methods, electricity price and CI, crude oil price, DAP price, anhydrous ammonia price, urea price, UAN price, labor wage adjustment ratio, targeted nitrogen fertilizer type, biocrude transportation distance, and hydrochar transportation distance.
2. Converting categorical variables using one-hot encoding into numerical representations.
3. Normalizing continuous features using StandardScaler, ensuring a mean of 0 and a standard deviation of 1 to precent bias from differing feature magnitudes.
4. Computing pairwise distances between data points using the Euclidean distance metric.
5. Initializing each data point as an individual cluster and iteratively merge the two closest clusters based on the Ward's linkage until 1,000 clusters were formed.
6. Calculating the median values of each continuous parameters in each cluster to reconstitute WRRFs. The median values were selected as they are robust to outliers and can reflect the central tendency for skewed distributions (e.g., wastewater solids mass flow rate, wastewater solids hauling distance).
7. Reconstructing 15,855 datapoints by replacing each original facility with the median values of parameters in its corresponding cluster, followed by calculating the coefficient of determination (R^2) between the original and reconstructed datasets. This resulted in an R^2 of 0.98 in this study, indicating the resulted 1,000 clusters preserved most of the variability in the original dataset.

4. Fisher's Z-test with Bonferroni correction

The sample size required for Spearman's correlation coefficients with α (significance threshold) = 0.05 and β (statistical power) = 0.2 was calculated through the following steps:

1. Converting Spearman's ρ into a normally distributed Z-score.

$$Z = 0.5\ln\left(\frac{1 + \rho}{1 - \rho}\right)$$

2. Adjusting α using Bonferroni correction based on the test number (n).

$$\alpha_{adjusted} = \frac{\alpha}{n}$$

3. Computing Z-score for $\alpha_{adjusted}$ using a two-tailed test (norm.ppf returns the Z-score corresponding to a given cumulative probability in a standard normal distribution).

$$Z_{\alpha_{adjusted}} = \text{norm.ppf}(1 - \frac{\alpha_{adjusted}}{2})$$

4. Computing Z-score for β.

$$Z_\beta = \text{norm.ppf}(\beta)$$

5. Computing the minimum samples size (N).

$$N = \left(\frac{Z_{\alpha_{adjusted}} + Z_\beta}{Z}\right)^2 + 3$$

S6. Consolidation of wastewater solids streams by HTL-based hubs

A k-d tree-based nearest neighbor search and graph algorithms were used to determine candidate HTL-based hub locations based on centroid positions through the following steps:

1. Determining the minimum wastewater solids mass flow rate to achieve net financial saving as 7 tonne·day^{-1} and the maximum average wastewater solids transportation distance to achieve net GHG reductions is 80 km (**Figures 5**, **S14-S16**). Both values were selected to be conservative to minimize false positives. The maximum transportation distance was converted to the maximum linear distance by dividing it by 1.3, which is the observed median ratio based on all transportation routes considered in this study. To assess the impact of this assumption, this ratio was adjusted to 1.1 (5th percentile) and 1.7 (95th percentile). However, the results showed minimal impacts on the covered wastewater solids ratio, though the hub count and covered WRRF count changed (**Table S15**). Therefore, only results using the median ratio (1.3) were reported in the primary analysis.
2. Employing k-d tree for efficient spatial indexing to reduce computational load by identifying candidate pairs of WRRFs.
3. Representing each WRRF as a node and adding an edge between two nodes in identified candidate pairs only if the distance was below a defined threshold. Repeating this step and identifying clusters of WRRFs.
4. Calculating the centroid of each cluster as a candidate hub location and computing the total wastewater solids mass flow rate and transportation distance from al WRRFs in the cluster to the centroid.
5. Removing candidate hubs based on whether they met predefined criteria in the step 1.

S7. Supplemental results

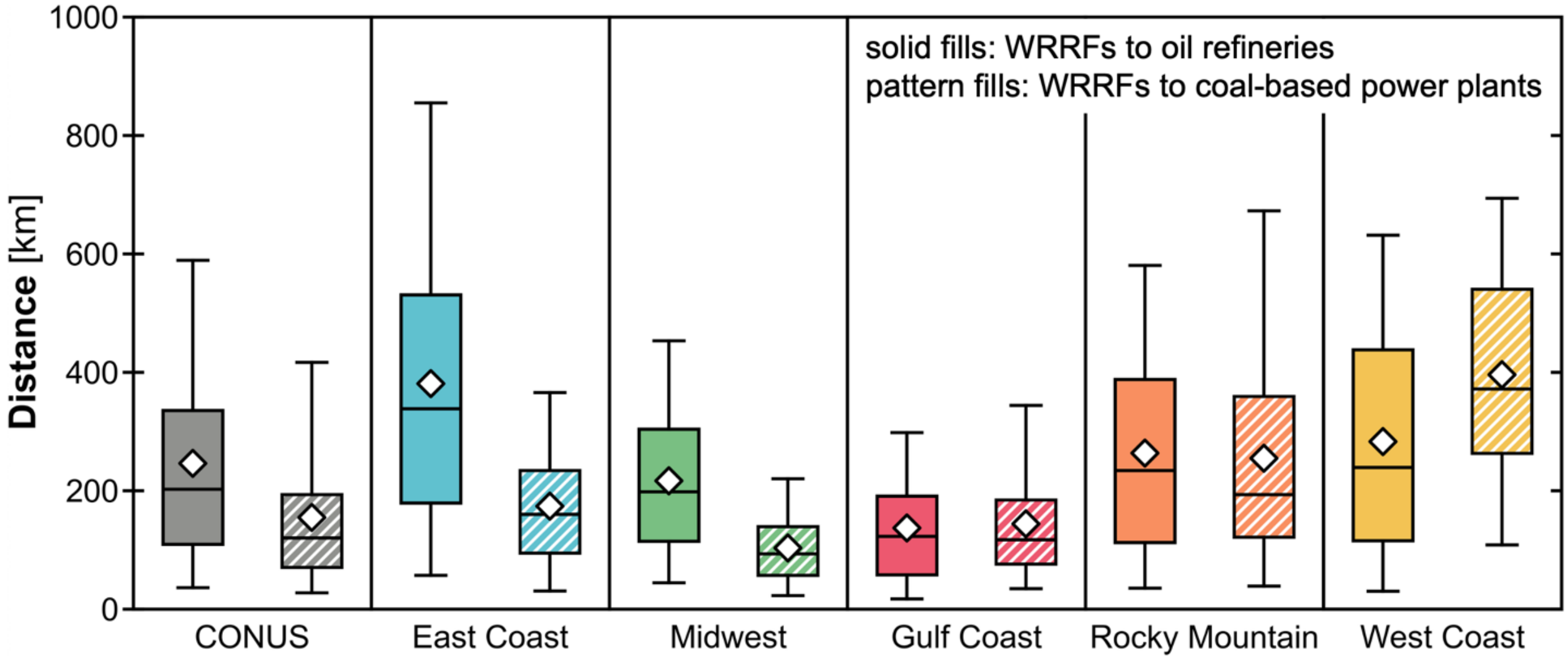

Figure S8. Travel distance from WRRFs in the contiguous U.S. (CONUS) or different CONUS regions to oil refineries (solid fills) and coal-based power plants (pattern fills). Whiskers, boxes, and midlines of the box plots represent 5th and 95th percentiles, 25th to 75th percentiles, and 50th percentiles of the travel distance, respectively. The diamonds represent average transportation distances.

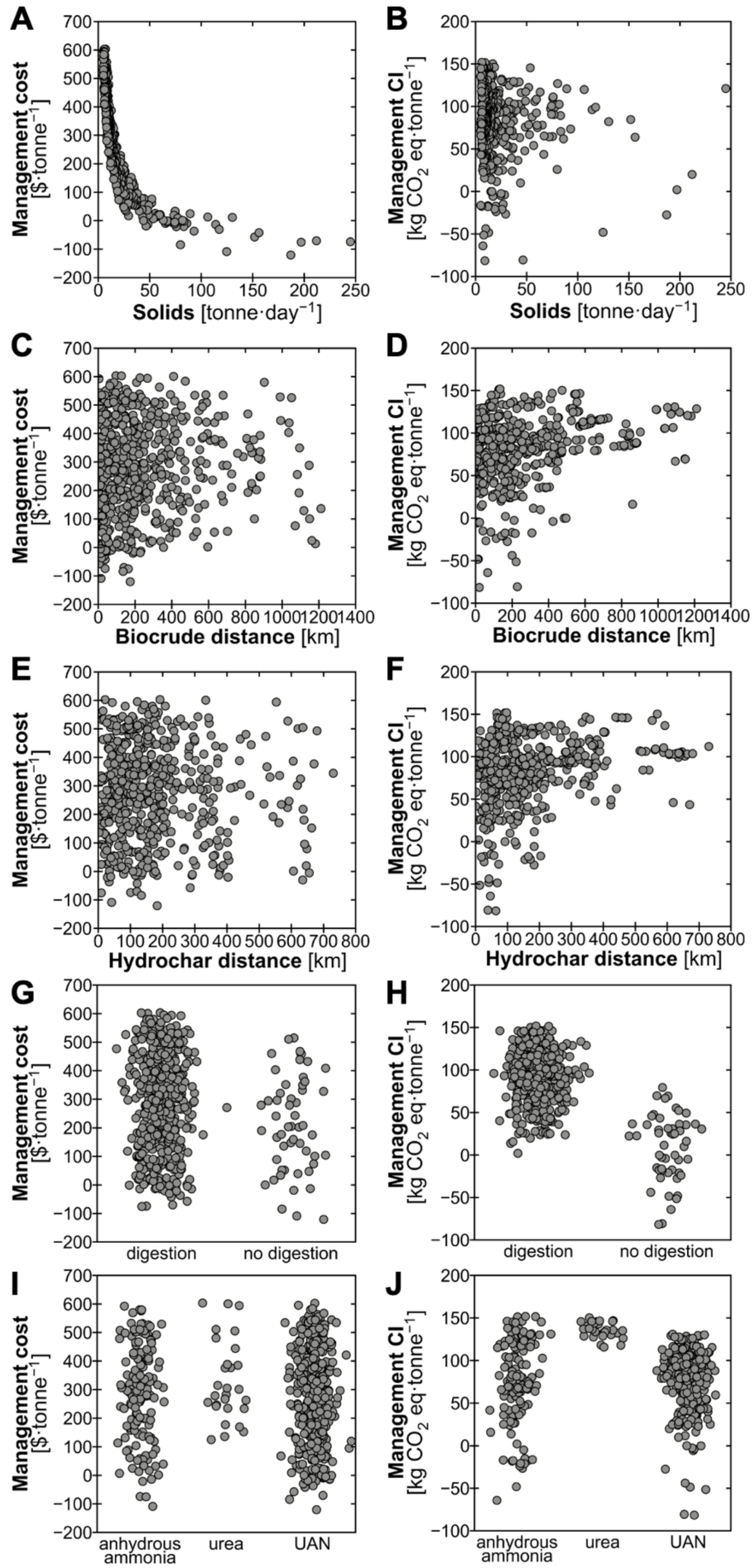

Figure S9. (A, C, E, G, I) Cost and (B, D, F, H, J) carbon intensity (CI) for HTL-based wastewater solids management as functions of (A, B) wastewater solids mass flow rate, (C, D) biocrude transportation distance, (E, F) hydrochar transportation distance, (G, H) the presence of wastewater solids digestion or not, and (I, J) the type of recovered nitrogen fertilizer. Each dot represents one WRRF.

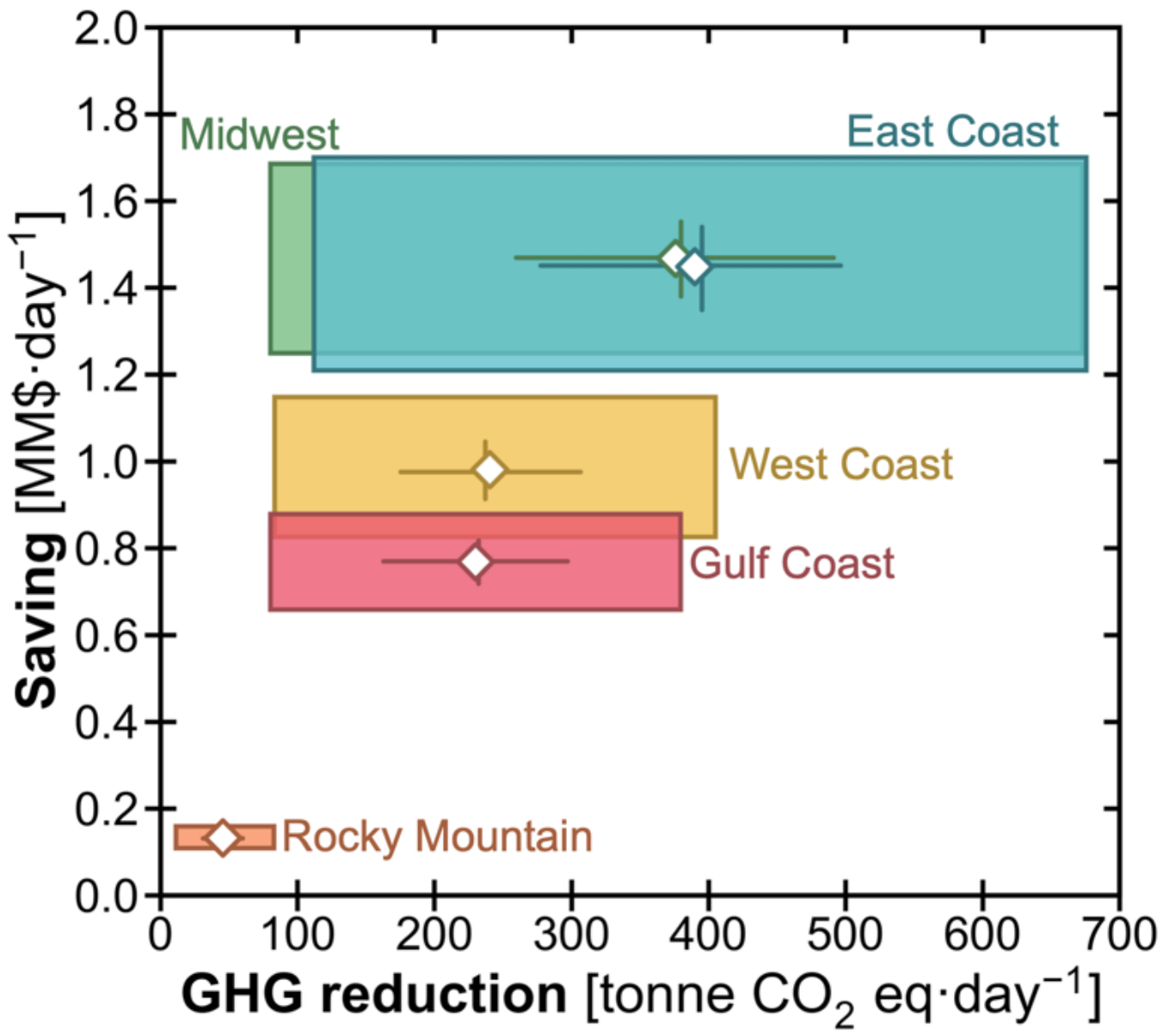

Figure S10. Saving and GHG reduction in different regions of the contiguous U.S. (blue: East Coast, green: Midwest, red: Gulf Coast, yellow: West Coast, orange: Rocky Mountain). The bottom boundary of the rectangles, the bottom end of the vertical lines, the diamonds, the upper end of the vertical lines, and the upper boundary of rectangles represent 5th, 25th, 50th, 75th, and 95th percentiles of saving under uncertainty, respectively. The left boundary of the rectangles, the left end of the horizontal lines, the white squares, the right end of the horizontal lines, and the right boundary of rectangles represent 5th, 25th, 50th, 75th, and 95th percentiles of GHG reduction amount under uncertainty, respectively. Uncertainty ranges are results of 1,000 Monte Carlo simulations.

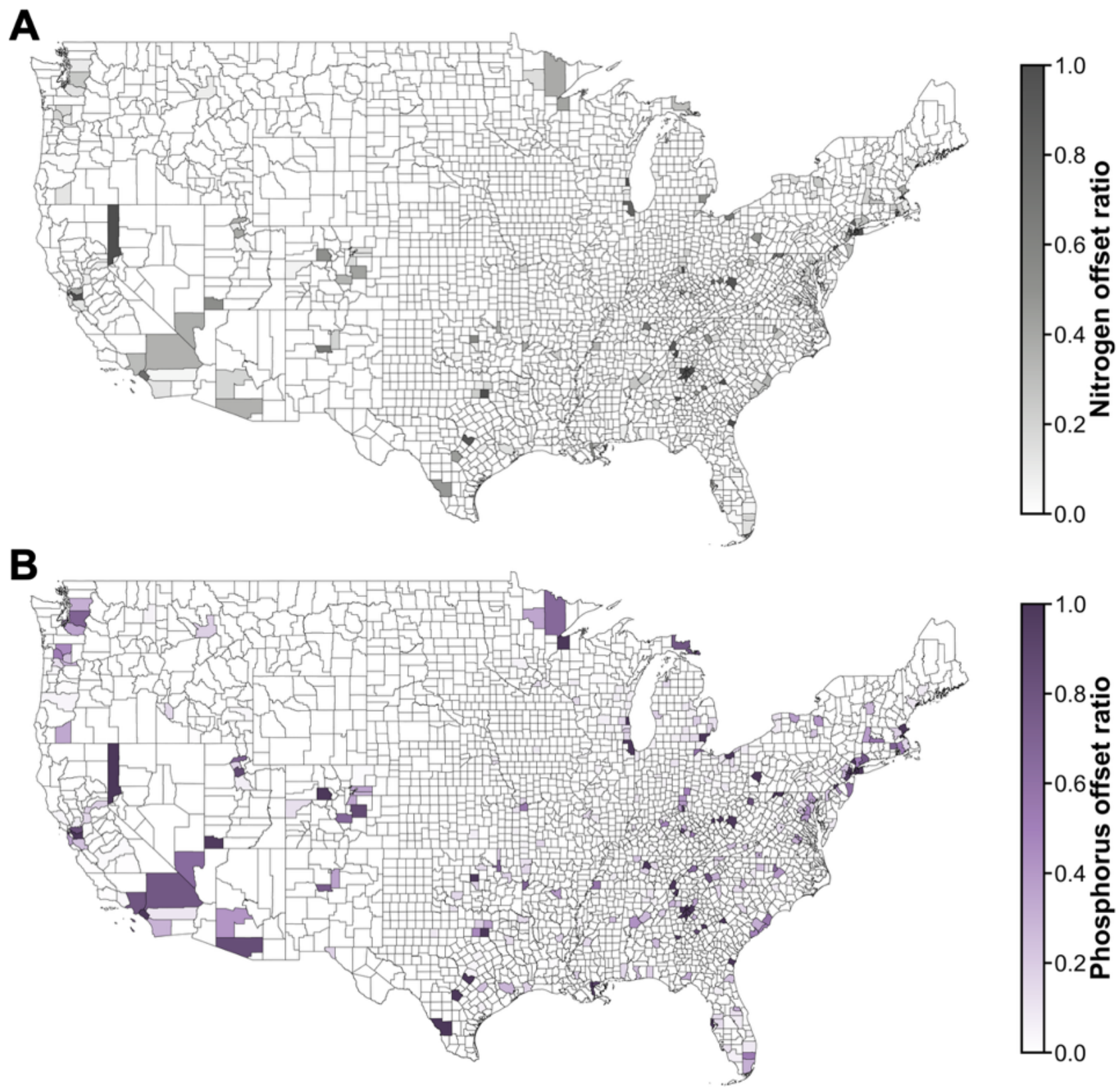

Figure S11. County-level (A) nitrogen and (B) phosphorus fertilizers offsets by products from HTL-based systems across the contiguous U.S., with the offset ratio capped at 1.

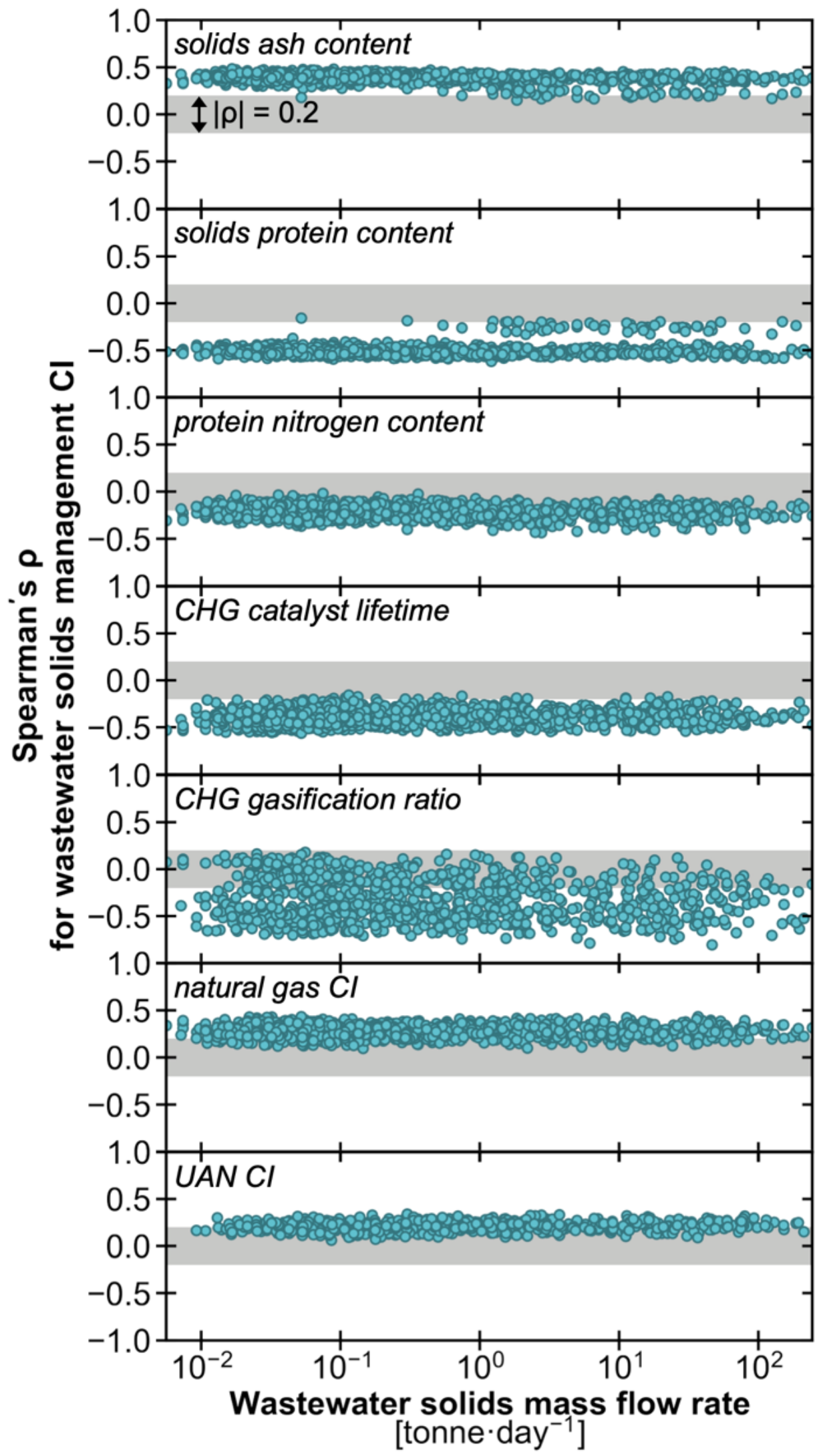

Figure S12. Spearman's ρ value of selected contextual parameters for 1,000 reconstituted WRRFs with different sizes (blue circles) on wastewater solids management CI. Parameters are selected if Spearman's $p < 0.05$ and the absolute value of Spearman's $\rho > 0.2$ for at least 10% of reconstituted WRRFs. Each Spearman's test is conducted based on 1,000 Monte Carlo simulations. Dashed lines represent Spearman's $\rho = \pm 0.2$. CI: carbon intensity, UAN: urea ammonium nitrate.

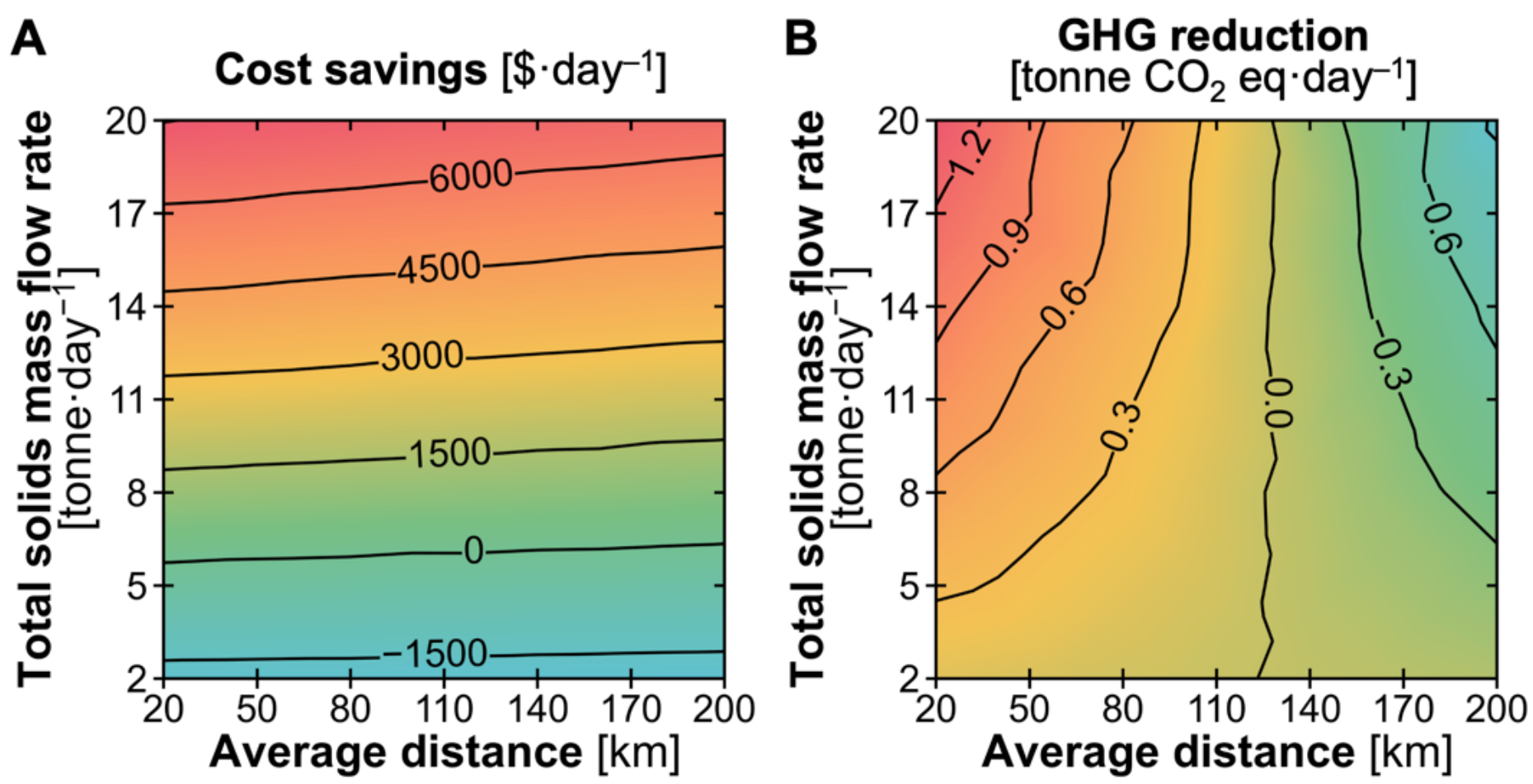

Figure S13. (A) Cost savings and (B) GHG reduction achieved by implementing HTL-based wastewater solids management systems (recovering urea ammonium nitrate, UAN) for 100 evenly-distributed combinations of total wastewater solids mass flow rate (from 2 to 20 tonne·day^{-1}) and average wastewater solids transportation distance (from 20 to 200 km). Each mass flow rate-distance combination was simulated with 1,000 Monte Carlo simulations, with median values shown in the figure.

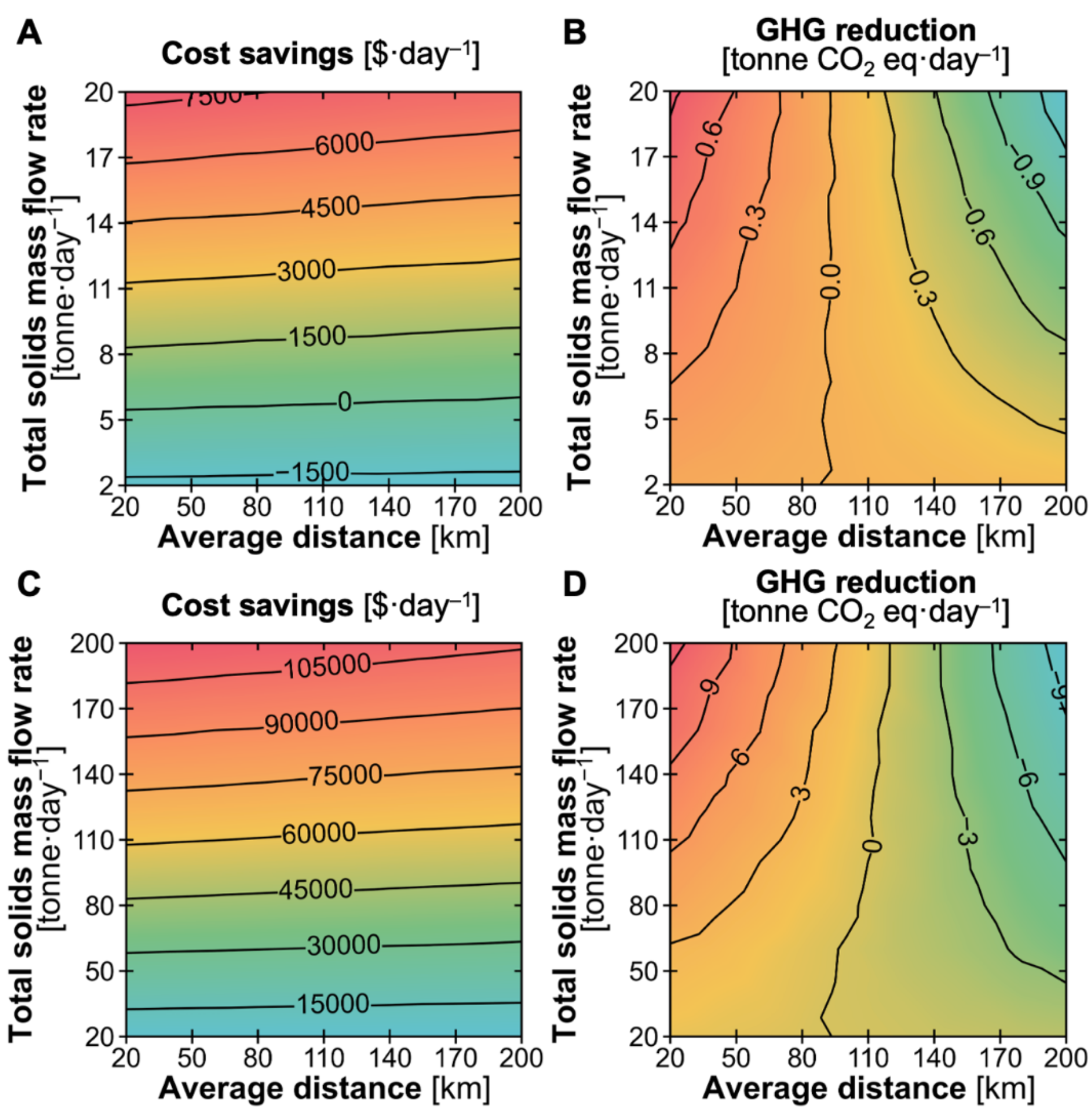

Figure S14. (A, C) Cost savings and (B, D) GHG reduction achieved by implementing HTL-based wastewater solids management systems (recovering anhydrous ammonia) for 100 evenly-distributed

combinations of total wastewater solids mass flow rate (A, B: from 2 to 20 tonne·day^{-1}, C, D: from 20 to 200 tonne·day^{-1}) and average wastewater solids transportation distance (from 20 to 200 km). Each mass flow rate-distance combination was simulated with 1,000 Monte Carlo simulations, with median values shown in the figure.

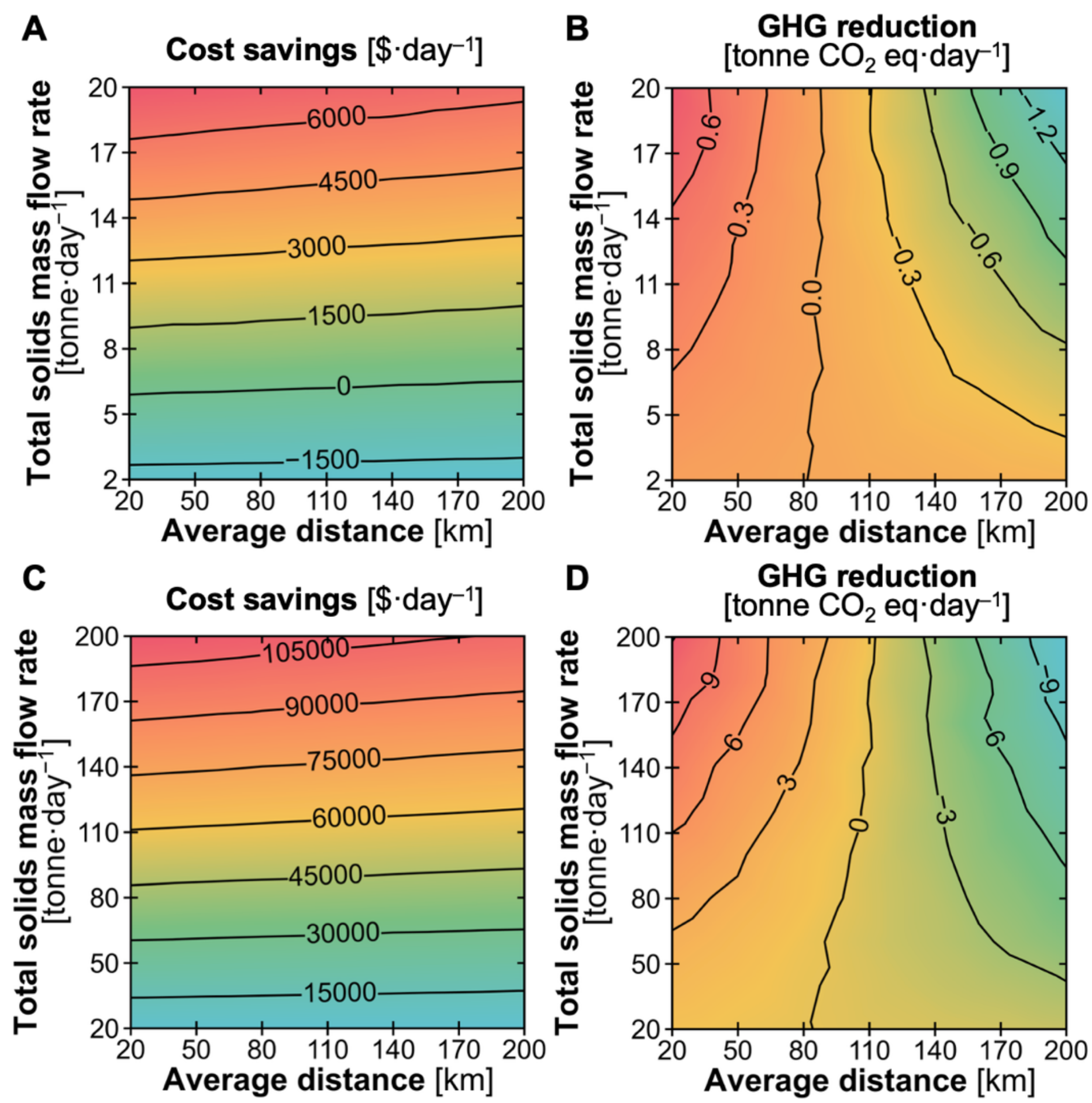

Figure S15. (A, C) Cost savings and (B, D) GHG reduction achieved by implementing HTL-based wastewater solids management systems (recovering urea) for 100 evenly-distributed combinations of total wastewater solids mass flow rate (A, B: from 2 to 20 tonne·day^{-1}, C, D: from 20 to 200 tonne·day^{-1}) and average wastewater solids transportation distance (from 20 to 200 km). Each mass flow rate-distance combination was simulated with 1,000 Monte Carlo simulations, with the median value of shown in the figure.

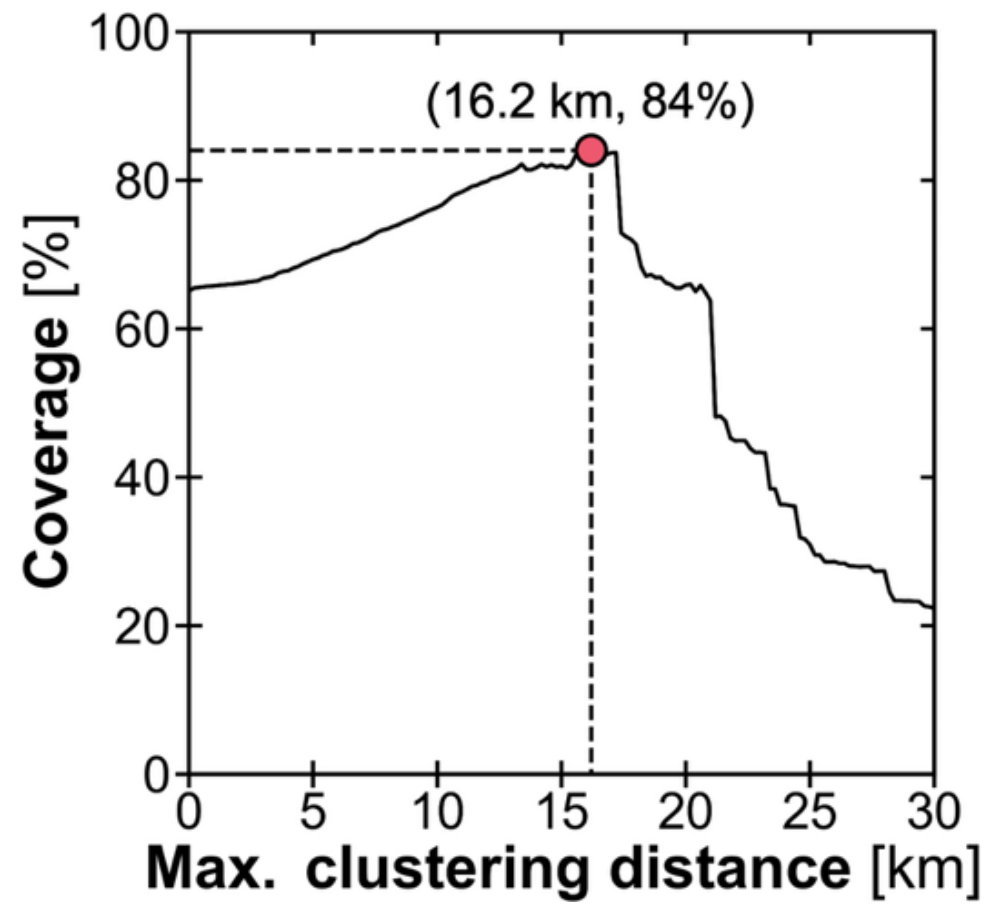

Figure S16. The coverage of wastewater solids by HTL-based hubs in the CONUS as a function of the maximum clustering distance used in k-d tree indexing. The maximum coverage (84%) occurs at a maximum clustering distance of 16.2 km.

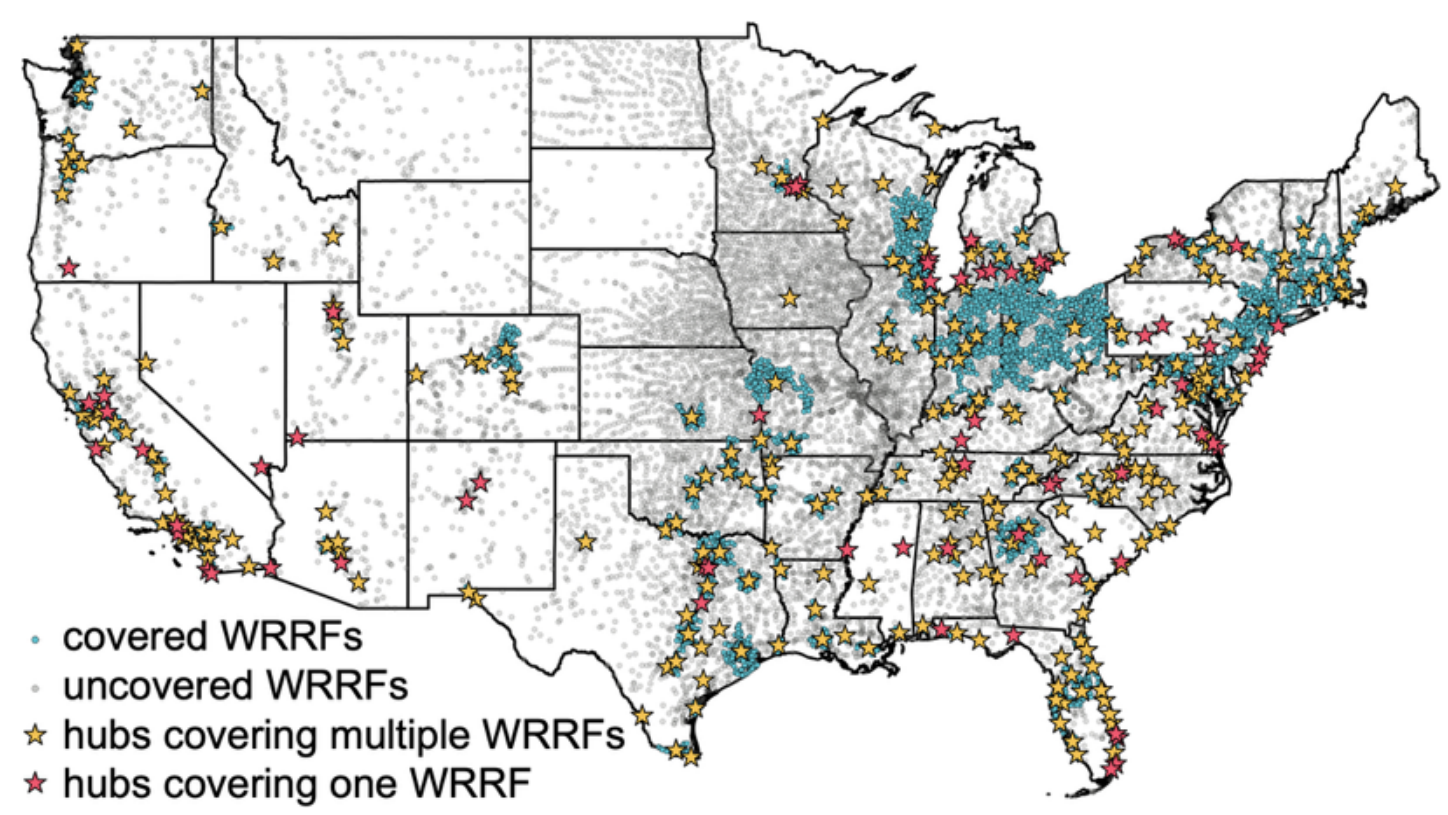

Figure S17. HTL-based hubs covering WRRFs across the CONUS. The maximum clustering distance of WRRFs for k-d tree indexing is 16.2 km. Blue and gray circles represent covered WRRFs and uncovered WRRFs, respectively. Yellow and red stars represent HTL-based hubs covering multiple WRRFs and one WRRF, respectively.

Table S9. Number of water resource recovery facilities (WRRFs) with different cost and carbon intensity (CI) reduction benefits by transitioning to HTL-based wastewater solids management.

benefits	WRRF count
cost reduction only	84
CI reduction only	15,111
cost and CI reductions	577 (576)[a]
no reduction	83

[a] One WRRF was excluded during the uncertainty analysis due to invalid results under extreme parameter combinations.

Table S10. Baseline capital expenditures (CAPEX) and operational expenditures (OPEX) comparisons between HTL-based systems with centralized (i.e., at oil refineries) and decentralized (i.e., with onsite hydrotreating and hydrocracking) biocrude upgrading at different wastewater solids mass flow rates. Simulations were run using the average wastewater solids biochemical composition, biocrude and hydrochar transportation distances, and other contextual parameters in the contiguous U.S., with the assumption of recovering urea nitrate ammonium (UAN).

**wastewater solids mass flow rate** [tonne·day^{-1}]	**centralized upgrading**		**decentralized upgrading**	
	**CAPEX** [$]	**OPEX** [$·year^{-1}]	**CAPEX** [$]	**OPEX** [$·year^{-1}]
0.000357 (min)	2,830,000	674,000	9,850,000	825,000
0.001	2,850,000	675,000	9,880,000	825,000
0.01	2,990,000	678,000	10,000,000	829,000
0.1	3,510,000	693,000	10,600,000	848,000
1	5,370,000	763,000	13,000,000	953,000
10	14,800,000	1,270,000	24,300,000	1,720,000
100	59,700,000	5,150,000	80,700,000	8,140,000
245 (max)	113,000,000	10,800,000	148,000,000	17,800,000

Table S11. Baseline carbon intensity (CI) breakdowns of HTL-based systems at different wastewater solids mass flow rates. Simulations were run using the average wastewater solids biochemical composition, biocrude and hydrochar transportation distances, and other contextual parameters in the contiguous U.S., with the assumption of recovering urea nitrate ammonium (UAN).

**wastewater solids mass flow rate** [tonne·day^{-1}]	**lifetime carbon intensity** [tonne CO_2 eq]				
	**chemical input**	**transportation**	**biofuel recovery**	**fertilizer recovery**	**electricity generation**
0.000357 (min)	1.58	0.049	−0.220	−1.03	−0.117
0.001	4.41	0.14	−0.616	−2.89	−0.327
0.01	44.10	1.40	−6.16	−28.9	−3.27
0.1	441	14.0	−61.6	−289	−32.7
1	4,410	140	−616	−2,890	−359
10	44,100	1,400	−6,160	−28,900	−4,010
100	441,000	14,000	−61,600	−289,000	−51,500
245 (max)	1,080,000	34,200	−151,000	−708,000	−150,000

Table S12. Relative importance of inter-WRRF contextual parameters on wastewater solids management cost and carbon intensity based on a Random Forest Regressor.

**Parameters**	**Management cost**	**Management CI**
wastewater solids mass flow rate	0.945	0.093
biocrude transportation distance	0.02	0.173
hydrochar transportation distance	0.0221	0.2
digestion	0.00222	0.262
no digestion	0.00224	0.166
recovering anhydrous ammonia	0.00581	0.0169
recovering urea	0.000546	0.0709
recovering UAN	0.00229	0.0174

Table S13. National aggregate results under different Gaussian copula coefficients.

results		Gaussian copula coefficient			
		0	0.5	0.8	0.999
phosphorus fertilizer offset	5th	2.1%	1.5%	1.4%	1.3%
	50th	2.2%	2.2%	2.1%	2.1%
	95th	2.3%	2.8%	3.0%	3.2%
nitrogen fertilizer offset	5th	0.9%	0.8%	0.7%	0.7%
	50th	1.0%	1.0%	1.0%	0.9%
	95th	1.0%	1.1%	1.2%	1.2%
biocrude production [BPD]	5th	21,400	18,700	18,100	17,800
	50th	21,600	21,700	21,600	21,500
	95th	21,900	24,200	24,900	25,600
saving [$·day^{-1}]	5th	4.75M	4.18M	4.04M	3.95M
	50th	4.80M	4.82M	4.81M	4.79M
	95th	4.85M	5.36M	5.51M	5.66M
CI reduction [tonne CO_2 eq·day^{-1}]	5th	1,220	532	351	241
	50th	1,290	1,320	1,300	1,280
	95th	1,360	1,970	2,140	2,320

Table S14. Baseline wastewater solids management cost [$·tonne^{-1}] at different internal rates of return (IRR) for varying wastewater solids mass flow rates. Simulations were run using the average wastewater solids biochemical composition, biocrude and hydrochar transportation distances, and other contextual parameters in the contiguous U.S., with the assumption of recovering urea nitrate ammonium (UAN). A higher average cost increasing rate [$·tonne^{-1}·IRR^{-1}] reflects greater sensitivity of costs to IRR.

**Solids mass flow rates** [tonne·day^{-1}]	**Median management cost at different IRR**						**Average cost increasing rate**
	**0%**	**1%**	**2%**	**3%**	**4%**	**5%**	
0.000357 (min)	6,010,000	6,130,000	6,280,000	6,440,000	6,590,000	6,760,000	14,900,000
0.001	2,150,000	2,190,000	2,250,000	2,300,000	2,360,000	2,420,000	5,360,000
0.01	217,000	222,000	227,000	233,000	239,000	245,000	562,000
0.1	22,400	22,900	23,500	24,200	24,900	25,600	65,300
1	2,360	2,440	2,540	2,640	2,750	2,850	9,850
10	213	234	261	289	318	347	2,680
100	−82.9	−74.4	−63.7	−52.3	−40.8	−29.2	1,070
245 (max)	−116	−110	−102	−92.7	−83.8	−74.8	831

Table S15. Hub analysis results using different transportation-to-linear distance ratio.

ratio	hub count	covered WRRF count	covered wastewater solids ratio
1.1	298	6,212	85%
1.3	329	5,692	84%
1.7	386	4,594	82%

References

(1) El Abbadi, S. H.; Feng, J.; Hodson, A. R.; Amouamouha, M.; Busse, M. M.; Polcuch, C.; Zhou, P.; Macknick, J.; Guest, J. S.; Stokes-Draut, J. R.; Dunn, J. B. Benchmarking Greenhouse Gas Emissions from U.S. Wastewater Treatment for Targeted Reduction. **2024**. https://doi.org/10.31223/X5VQ59.

(2) U.S. Environmental Protection Agency. Clean Watersheds Needs Survey 2004, 2008. https://www.epa.gov/cwns.

(3) U.S. Environmental Protection Agency. Clean Watersheds Needs Survey 2008, 2010. https://www.epa.gov/cwns.

(4) U.S. Environmental Protection Agency. Clean Watersheds Needs Survey 2012, 2016. https://www.epa.gov/cwns.

(5) U.S. Environmental Protection Agency. Clean Watersheds Needs Survey 2022, 2024. https://www.epa.gov/cwns.

(6) U.S. Environmental Protection Agency. *Biosolids Biennial Report No. 9 (Reporting Period 2020-2021)*; EPA-822R22007; Office of Science & Technology: Washington, DC, 2022. https://www.epa.gov/system/files/documents/2022-12/2020-2021-biennial-report.pdf.

(7) U.S. Department of Energy (DOE). U.S. Department of Energy Combined Heat and Power Installation Database, 2024. https://doe.icfwebservices.com/chp (accessed 2018-08-12).

(8) U.S. Energy Information Administration (EIA). *Refinery Capacity Report*. https://www.eia.gov/petroleum/refinerycapacity/index.php (accessed 2024-08-27).

(9) Falcone, J. A. *Estimates of County-Level Nitrogen and Phosphorus from Fertilizer and Manure from 1950 through 2017 in the Conterminous United States*; 2020–1153; U.S. Geological Survey, 2021. https://doi.org/10.3133/ofr20201153.

(10) Cao, P.; Lu, C.; Yu, Z. Historical Nitrogen Fertilizer Use in Agricultural Ecosystems of the Contiguous United States during 1850–2015: Application Rate, Timing, and Fertilizer Types. *Earth Syst. Sci. Data* **2018**, *10* (2), 969–984. https://doi.org/10.5194/essd-10-969-2018.

(11) *USDA ERS - Fertilizer Use and Price*. https://www.ers.usda.gov/data-products/fertilizer-use-and-price/ (accessed 2024-12-24).

(12) *Electric Power Annual - U.S. Energy Information Administration (EIA)*. https://www.eia.gov/electricity/annual/index.php (accessed 2024-08-27).

(13) Gagnon, P.; Frazier, W.; Cole, W.; Schwarz, M.; Hale, E. *Cambium Data for 2021 Standard Scenarios*; National Renewable Energy Laboratory. https://cambium.nrel.gov/.

(14) *Domestic Crude Oil First Purchase Prices by Area*. https://www.eia.gov/dnav/pet/pet_pri_dfp1_k_m.htm (accessed 2025-02-24).

(15) *USDA Agricultural Marketing Service*. https://mymarketnews.ams.usda.gov/report/list?market_types%5B0%5D=214 (accessed 2025-02-24).

(16) *County Employment and Wages Full Data Update*. Bureau of Labor Statistics. https://www.bls.gov/web/cewdat.supp.toc.htm (accessed 2025-02-24).

(17) Fritts, J. *State Corporate Income Tax Rates and Brackets for 2022*. Tax Foundation. https://taxfoundation.org/data/all/state/state-corporate-income-tax-rates-brackets-2022/ (accessed 2025-02-24).

(18) *QSD-Group/EXPOsan at bd1c52aee0019b891e8aa647f434b30f356148c1*. GitHub. https://github.com/QSD-Group/EXPOsan (accessed 2025-08-15).

(19) *QSD-Group/QSDsan at 988a233a3129f1519e8d3519e4bdfb1f29f2ae6a*. GitHub. https://github.com/QSD-Group/QSDsan (accessed 2025-08-14).

(20) *BioSTEAMDevelopmentGroup/biosteam at 2da303976fee0dadd4ba5ae1f71c41d42370c6b8*. GitHub. https://github.com/BioSTEAMDevelopmentGroup/biosteam (accessed 2025-07-31).

(21) Feng, J.; Li, Y.; Strathmann, T. J.; Guest, J. S. Characterizing the Opportunity Space for Sustainable Hydrothermal Valorization of Wet Organic Wastes. *Environ. Sci. Technol.* **2024**, *58* (5), 2528–2541. https://doi.org/10.1021/acs.est.3c07394.

(22) Snowden-Swan, L. J.; Li, S.; Thorson, M. R.; Schmidt, A. J.; Cronin, D. J.; Zhu, Y.; Hart, T. R.; Santosa, D. M.; Fox, S. P.; Lemmon, T. L.; Swita, M. S. *Wet Waste Hydrothermal Liquefaction and Biocrude Upgrading to Hydrocarbon Fuels: 2022 State of Technology*; PNNL-33622; Pacific Northwest National Lab. (PNNL), Richland, WA (United States), 2022. https://doi.org/10.2172/1897670.

(23) Geopandas: Geographic Pandas Extensions.

(24) Googlemaps: Python Client Library for Google Maps Platform. https://github.com/googlemaps/google-maps-services-python (accessed 2023-09-18).

(25) Seider, W. D.; Lewin, D. R.; Seader, J. D.; Widagdo, S.; Gani, R.; Ng, K. M. *Product and Process Design Principles*, Fourth Edition.; Wiley, 2017.

(26) Devkota, S.; Karmacharya, P.; Maharjan, S.; Khatiwada, D.; Uprety, B. Decarbonizing Urea: Techno-Economic and Environmental Analysis of a Model Hydroelectricity and Carbon Capture Based Green Urea Production. *Appl. Energy* **2024**, *372*, 123789. https://doi.org/10.1016/j.apenergy.2024.123789.

(27) Palys, M. J.; Daoutidis, P. Techno-Economic Optimization of Renewable Urea Production for Sustainable Agriculture and CO2 Utilization. *J. Phys. Energy* **2023**, *6* (1), 015013. https://doi.org/10.1088/2515-7655/ad0ee6.

(28) US EPA, O. *Lifecycle Analysis of Greenhouse Gas Emissions under the Renewable Fuel Standard*. https://www.epa.gov/renewable-fuel-standard-program/lifecycle-analysis-greenhouse-gas-emissions-under-renewable-fuel (accessed 2024-08-30).

(29) Peccia, J.; Westerhoff, P. We Should Expect More out of Our Sewage Sludge. *Environ. Sci. Technol.* **2015**, *49* (14), 8271–8276. https://doi.org/10.1021/acs.est.5b01931.

(30) *Cost of Landspreading and Hauling Sludge from Municipal Wastewater Treatment Plants Case Studies*; EPA/530/SW-619; United States Environmental Protection Agency, 1977.

(31) Fritts, J. *State Corporate Income Tax Rates and Brackets for 2023*. Tax Foundation. https://taxfoundation.org/data/all/state/state-corporate-income-tax-rates-brackets-2023/ (accessed 2023-12-27).

(32) Stewart, D. W.; Cortés-Peña, Y. R.; Li, Y.; Stillwell, A. S.; Khanna, M.; Guest, J. S. Implications of Biorefinery Policy Incentives and Location-Specific Economic Parameters for the Financial Viability of Biofuels. *Environ. Sci. Technol.* **2023**. https://doi.org/10.1021/acs.est.2c07936.

(33) *ECI Home*. Bureau of Labor Statistics. https://www.bls.gov/eci/ (accessed 2024-09-01).

(34) Snowden-Swan, L. J.; Zhu, Y.; Bearden, M. D.; Seiple, T. E.; Jones, S. B.; Schmidt, A. J.; Billing, J. M.; Hallen, R. T.; Hart, T. R.; Liu, J.; Albrecht, K. O.; Fox, S. P.; Maupin, G. D.; Elliott, D. C. *Conceptual Biorefinery Design and Research Targeted for 2022: Hydrothermal*

Liquefacation Processing of Wet Waste to Fuels; PNNL-27186; Pacific Northwest National Lab. (PNNL), Richland, WA (United States), 2017. https://doi.org/10.2172/1415710.

(35) Leow, S.; Shoener, B. D.; Li, Y.; DeBellis, J. L.; Markham, J.; Davis, R.; Laurens, L. M. L.; Pienkos, P. T.; Cook, S. M.; Strathmann, T. J.; Guest, J. S. A Unified Modeling Framework to Advance Biofuel Production from Microalgae. *Environ. Sci. Technol.* **2018**, *52* (22), 13591–13599. https://doi.org/10.1021/acs.est.8b03663.

(36) Ecoinvent 3.8 Database. Swiss Centre for Life Cycle Inventories (accessed 2023-01-06).

(37) Bare, J. TRACI 2.0: The Tool for the Reduction and Assessment of Chemical and Other Environmental Impacts 2.0. *Clean Technol. Environ. Policy* **2011**, *13* (5), 687–696. https://doi.org/10.1007/s10098-010-0338-9.

(38) IPCC. *Climate Change 2022: Impacts, Adaptation, and Vulnerability. Contribution of Working Group II to the Sixth Assessment Report of the Intergovernmental Panel on Climate Change*; Pörtner, H.-O., Roberts, D. C., Tignor, M., Poloczanska, E. S., Mintenbeck, K., Alegría, A., Craig, M., Langsdorf, S., Löschke, S., Möller, V., Okem, A., Rama, B., Series Eds.; IPCC: Cambridge, UK and New York, NY, USA, 2022. https://doi.org/10.1017/9781009325844.

(39) Li, Y.; Leow, S.; Fedders, A. C.; Sharma, B. K.; Guest, J. S.; Strathmann, T. J. Quantitative Multiphase Model for Hydrothermal Liquefaction of Algal Biomass. *Green Chem.* **2017**, *19* (4), 1163–1174. https://doi.org/10.1039/C6GC03294J.
